# Genomic insights into divergence and dual domestication of cultivated allotetraploid cottons

**DOI:** 10.1186/s13059-017-1167-5

**Published:** 2017-02-20

**Authors:** Lei Fang, Hao Gong, Yan Hu, Chunxiao Liu, Baoliang Zhou, Tao Huang, Yangkun Wang, Shuqi Chen, David D. Fang, Xiongming Du, Hong Chen, Jiedan Chen, Sen Wang, Qiong Wang, Qun Wan, Bingliang Liu, Mengqiao Pan, Lijing Chang, Huaitong Wu, Gaofu Mei, Dan Xiang, Xinghe Li, Caiping Cai, Xiefei Zhu, Z. Jeffrey Chen, Bin Han, Xiaoya Chen, Wangzhen Guo, Tianzhen Zhang, Xuehui Huang

**Affiliations:** 1 State Key Laboratory of Crop Genetics and Germplasm Enhancement, Cotton Hybrid R & D Engineering Center (the Ministry of Education), Nanjing Agricultural University, Nanjing, 210095 China; 2 National Center for Gene Research, Institute of Plant Physiology and Ecology, Shanghai Institutes for Biological Sciences, Chinese Academy of Sciences, Shanghai, 200233 China; 3 Cotton Fiber Bioscience Research Unit, USDA-ARS-SRRC, New Orleans, LA 70124 USA; 4 State Key Laboratory of Cotton Biology, Institute of Cotton Research of the Chinese Academy of Agricultural Sciences, Anyang, China; 5 Cotton Research Institute, Xinjiang Academy of Agriculture and Reclamation Sciences, Xinjiang, 832000 China; 6 Department of Molecular Biosciences, Center for Computational Biology and Bioinformatics, and Institute for Cellular and Molecular Biology, the University of Texas at Austin, Austin, TX 78712 USA; 7 State Key Laboratory of Plant Molecular Genetics, Institute of Plant Physiology and Ecology, Shanghai Institutes for Biological Sciences, Chinese Academy of Sciences, Shanghai, 200032 China; 8 Agronomy Department, College of Agriculture and Biotechnology, Zhejiang University, Zhejiang, 310029 China; 9 College of life and environmental sciences, Shanghai Normal University, Shanghai, 200234 China

**Keywords:** Allotetraploid cottons, Resequencing, Divergence, Domestication

## Abstract

**Background:**

Cotton has been cultivated and used to make fabrics for at least 7000 years. Two allotetraploid species of great commercial importance, *Gossypium hirsutum* and *Gossypium barbadense*, were domesticated after polyploidization and are cultivated worldwide. Although the overall genetic diversity between these two cultivated species has been studied with limited accessions, their population structure and genetic variations remain largely unknown.

**Results:**

We resequence the genomes of 147 cotton accessions, including diverse wild relatives, landraces, and modern cultivars, and construct a comprehensive variation map to provide genomic insights into the divergence and dual domestication of these two important cultivated tetraploid cotton species. Phylogenetic analysis shows two divergent groups for *G. hirsutum* and *G. barbadense*, suggesting a dual domestication processes in tetraploid cottons. In spite of the strong genetic divergence, a small number of interspecific reciprocal introgression events are found between these species and the introgression pattern is significantly biased towards the gene flow from *G. hirsutum* into *G. barbadense*. We identify selective sweeps, some of which are associated with relatively highly expressed genes for fiber development and seed germination.

**Conclusions:**

We report a comprehensive analysis of the evolution and domestication history of allotetraploid cottons based on the whole genomic variation between *G. hirsutum* and *G. barbadense* and between wild accessions and modern cultivars. These results provide genomic bases for improving cotton production and for further evolution analysis of polyploid crops.

**Electronic supplementary material:**

The online version of this article (doi:10.1186/s13059-017-1167-5) contains supplementary material, which is available to authorized users.

## Background

Cotton (*Gossypium* spp.) is the most important natural fiber and edible oil crop in the world. The genus *Gossypium* includes around 45 diploid (2n = 2x = 26) and five allotetraploid (2n = 4x = 52) species. The allotetraploids that were present 1–1.5 million years ago (MYA) originated from one hybridization event between an extant progenitor of *Gossypium herbaceum* (A_1_) or *Gossypium arboreum* (A_2_) and another progenitor, *Gossypium raimondii* Ulbrich (D_5_) [1–3]. *Gossypium* wild relatives grew primarily as perennial upright shrubs or small trees and existed in various stages of domestication as feral derivatives that had established self-perpetuating populations in human-modified environments such as road sides, field edges, and dooryards [4]. Cotton is a unique example of crop domestication that occurred in two Old World diploids, *G. herbaceum* L. and *G. arboreum* L. and two New World allotetraploids, *Gossypium hirsutum* and *Gossypium barbadense*, in four different pre-historical cultures [4]. Under long-term human selection of a wide range of morphological and physiological traits, the two tetraploid species, *G. hirsutum* and *G. barbadense*, have been domesticated and cultivated. However, photoperiod sensitivity in long-lived perennial species with a slow rate of plant development and seed emergence and the broad spectrum of fruiting habits in cultivars have been under investigated [5–7].

Modern *G. hirsutum* cultivars (Upland cotton) have high-yield properties and dominate more than 90% of worldwide cotton production, while *G. barbadense*, characterized by its extra-long staple (ELS) and strong and fine fibers accounts for less than 10% [8]. *G. hirsutum* is native to the Mesoamerican and the Caribbean regions, and *G. barbadense* is indigenous to the coastal areas of Peru [9, 10]. Through intensive study of germplasm collections, Hutchinson [11] identified one wild and six domesticated (not botanical varieties) races of *G. hirsutum* based mainly on their morphologies and distinct geographic distributions. Modern Upland cotton has been further improved in the southern United States from domesticated early-cropping perennials through extensive human selection to produce a common set of agronomic features known as “domestication syndrome” traits [12]. These traits include an annual growth habit and photoperiod insensitivity [5], decreased seed dormancy [6], a large boll size and number per plant [1], and superior fiber quality [13]. The genetic diversity of allotetraploid cottons has been studied for decades using pedigree information and morphologies [14, 15], biochemical markers [7, 16], and DNA-based markers [17–20]. Genomic insights into variation within and between allotetraploid cotton species are limited by the lack of known allotetraploid genome sequences. To resolve this, we resequenced and conducted genomic analysis of 147 cotton accessions with different origins after sequencing the genome of the genetic standard Upland cotton line, TM-1 [21]. Until now, only a few candidate genes related to cotton lint yield and fiber quality have been functionally characterized. So, we integrated the expression profiling data, quantitative trait loci (QTL) mapping, and function annotations with orthologs in *Arabidopsis* to conduct rapid identification of genes associated with domestication, especially fiber development and seed germination. The present research provides genome-wide level insights into genetic divergence and dual domestication of cultivated tetraploid cottons.

## Results and discussion

### Genetic diversity

Upland and Sea Island varieties were established in the seaboard colonies of the southeastern United States by the mid-18th century and the Egyptian cottons in the Nile Delta by the early 19th century. So, we sampled 147 *G. hirsutum* and *G. barbadense* accessions, including wild species, races, landraces, and modern improved cultivars, from different geographic locations, representing the long history of cotton domestication and breeding throughout the world (Table 1; Additional file 1: Figure S1; Additional file 2: Table S1). Close relatives of the allotetraploid cotton species, *Gossypium tomentosum* (AD)_3_, *Gossypium mustelinum* (AD)_4_, and *Gossypium darwinii* (AD)_5_, as well as *Thespesia populneoides* (Roxb.) Kostelas, which is closely related to the genus *Gossypium* in the Malvaceae family, were all included as outgroups. We resequenced all 147 accessions with approximately fivefold coverage, generating a total of 1.8 terabases of raw sequence data, and aligned the reads to the reference genome sequence of TM-1 [21] to identify sequence variants (Table 1). We used direct genome sequence comparison and PCR-based sequencing strategies to validate the quality of the called single nucleotide polymorphisms (SNPs). Two recently sequenced accessions of *G. barbadense* cv. Xinhai 21 (XH21) [22] and *G. hirsutum* acc.TM-1 [21] in our sequence panel were used as controls. We checked the called SNPs from our sequence panel against two assembled genome sequences and found the accuracy of SNP calling to be 96.2% for XH21 and 99.1% for TM-1, with a low missing data rate (6.8%). We further randomly selected 68 SNPs to carry out PCR-based sequencing in 11 accessions, each randomly selected from one cluster of the phylogenetic tree constructed with 147 accessions, and found that the accuracy was 95.0% (Additional file 1: Figure S2; Additional file 3: Table S2; Additional file 4: Table S3). Therefore, the quality should be reliable enough for follow-up phylogenetic and population genetic analyses.

**Table 1:** Summary of sequencing of and variations in *G. hirsutum* and *G. barbadense*

Group	Accessions (number)	Raw data (Gb)	Raw data depth	Uniquely mapping rate to the A subgenome	Uniquely mapping rate to the D subgenome
*G. hirsutum* cultivars	52	734	5.64	36.1%	24.9%
*G. hirsutum* races	33	442	5.35	34.3%	22.4%
*G. barbadense* cultivars	52	534	4.10	36.6%	24.3%
Others^a^	10	132	5.19	35.2%	21.0%
Total	147	1,842	5.17	36.2%	23.9%

^a^ *Others* includes four *G. barbadense* races, Kaiyuanlihemumian, Yuanmoulihemumian, Alabolihemumian, and Kaiyuanlianhemumian, and close relatives *Thespesia populneoides* (Roxb.) Kostel, *Gossypium purpurascens*, *G. mustelinum*, *G. darwinii*, and an Indian cultivar, NV50-70

Of the sequenced reads, 36.2 and 23.9% were uniquely mapped to the A and D subgenomes of the TM-1 reference genome (1.9-Gb oriented scaffold), respectively (Table 1). Additionally, 10.5% of the total reads were mapped to the A subgenome unoriented scaffolds and 1.9% of the total reads were mapped to the D subgenome unoriented scaffolds; we did not use these A or D subgenome unoriented scaffolds for further analysis. Moreover, 23.4% of the total reads were mapped to no or multiple locations, which may be caused by the high proportion of repeated sequence (67.2%) or the highly homoeologous regions between the A and D subgenome in cotton. Only 4.1% of the total reads were mapped to the unclassified scaffolds, which had little effect on our analysis.

Overall, we identified 16,377,749 non-unique SNPs, defined as those with the variant occurring in at least two accessions and 144,662 non-unique indels (1 bp–8 kb; Additional file 5: Table S4). Of these indels, 16,879 with >50-bp indels were identified as structural variants (SVs; Additional file 1: Figure S3; Additional file 6: Table S5). For instance, the SV (2992 bp) identified in chromosome D09 from 44,118,172 to 44,121,164 bp could be detected in 37 accessions. These variants were distributed across all 26 chromosomes, with an average density of 8.5 SNPs per kilobase (Additional file 7: Table S6). The SNP density in the A subgenome (9.2 SNPs per kilobase) was higher than that in the D subgenome (7.4 SNPs per kilobase). By analyzing the allele frequency of each SNP site in the 147 accessions, we identified 7,993,856 common SNPs, each with an allele frequency of >5%, including 3,203,112 intraspecific SNPs in *G. hirsutum*, 3,770,221 in *G. barbadense*, and 2,752,128 (~34.4%) nearly fixed interspecific SNPs (SNPs with an allele frequency of >95% in *G. hirsutum* or *G. barbadense* and <5% in the other species (Additional file 1: Figure S4).

### Dual domestication of cultivated allotetraploid cottons

**Fig. 1:**
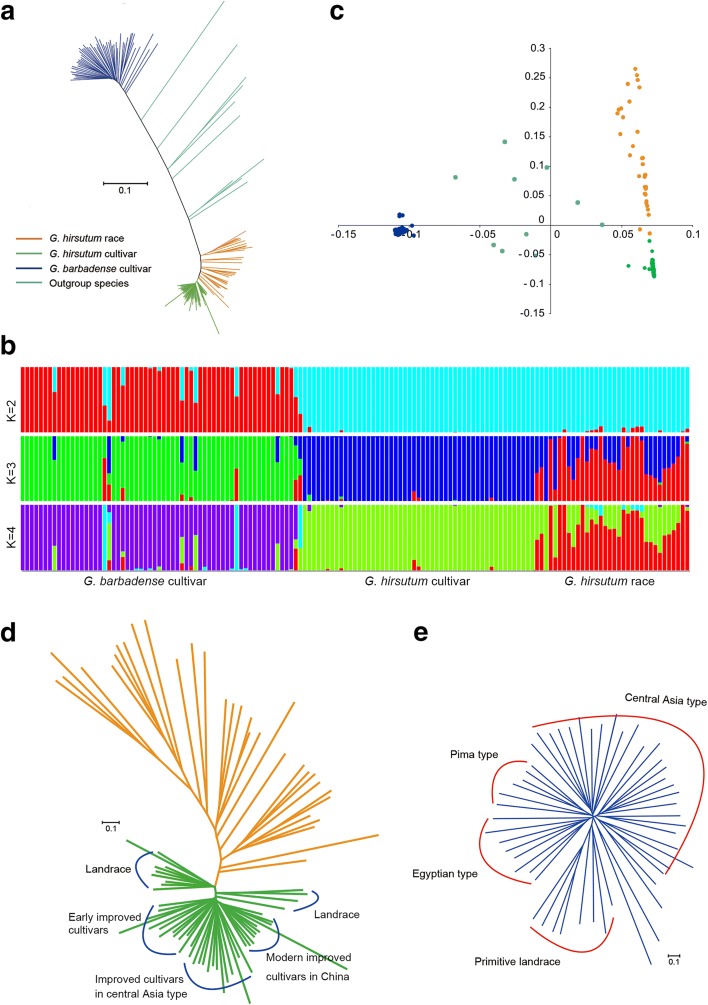
Phylogenetic relationships of 147 cotton accessions. **a** A neighbor-joining tree was constructed using whole-genome SNP data. The cotton samples were divided into *G. hirsutum* races (*orange*), *G. hirsutum* cultivars (*green*), *G. barbadense* cultivars (*dark blue*) and outgroup species (*light blue*). **b** Population structure of cotton accessions determined using STRUCTURE. The accessions were divided into three groups when K = 3. **c** Principal component analysis of all cotton accessions using whole-genome SNP data. **d** Phylogenetic relationships between *G. hirsutum* cultivars and races. **e** Phylogenetic relationships between *G. barbadense* landraces and cultivars. The *scale bar* indicates the simple matching distance

The whole-genome SNP data were used to investigate the phylogenetic relationships between all allotetraploid cotton collections (Fig. 1a; Additional file 8: Dataset 1). The subsequently produced neighbor-joining (NJ) tree resulted in two largely divergent clades: the *G. hirsutum* clade (*n* = 85) and the *G. barbadense* clade (*n* = 52), consistent with a previous study, although with a limited number of accessions [23]. Both studies suggested a strong divergence between *G. hirsutum* and *G. barbadense*. Model-based analyses of population structure using STRUCTURE revealed that there were two different components between *G. barbadense* and *G. hirsutum* when K (the number of populations modeled) was set to 2. However, when K was set to 3, there were three different components: *G. barbadense* cultivars, *G. hirsutum* cultivars, and *G. hirsutum* races (Fig. 1b). This model-based result, along with that from principal component analysis (Fig. 1c), agreed well with the pattern in the phylogenetic tree. The outgroup type comprised ten accessions in total, including *T. populneoides*, *G. tomentosum* (Hawaiian Islands), *G. darwinii* (Galapagos Islands), and seven tetraploid accessions that might have resulted from genetic introgressions from wild progenitors or from historical interspecific crossing between *G. hirsutum* and *G. barbadense* (Additional file 2: Table S1). No clear separation existed between the seven races (33 accessions in total) in *G. hirsutum*, which was likely due to human-mediated accession expansion, bringing formerly isolated races into mixed and overlapping distributions (Fig. 1d). However, one punctatum race from Egypt and one latifolium race from Chiapas were most closely related to *G. hirsutum* cultivars (Fig. 1; Additional file 8: Dataset 1). Some African and Indian cultivars were classified into one landrace subgroup, which was closely related to the true annual forms of punctatum grown in Africa, further supporting the early cropping of race punctatum in the Old World [11]. Punctatum is a race originally found inland on the Yucatan peninsula. Whether annual forms of punctatum were developed before or after its introduction into Africa remains to be explored. These genomic data revealed at least two origins of upland cotton in the Old World and the New World; punctatum in America or Africa and latifolium in America, consistent with the domestication and improvement history of upland cotton [1, 2, 11, 18].

The origins of modern cultivated *G. barbadense* are complex and somewhat obscure. Unlike *G. hirsutum*, which exists in both wild and cultivated states, *G. barbadense* is found only in cultivars. The present research provides genomic evidence that *G. barbadense* is indigenous to Peru and Brazil since those primitive landraces of *G. barbadense* native to Brazil and Peru together with West of the Andes and Sea Island cotton were classified into one subgroup (Fig. 1e). It suggests a probable center of origin in northwestern South America, consistent with archeological records [24]. All modern ELS cultivars were classified into three subgroups: Egyptian, American Pima, and Central Asia cottons.

### Genomic divergence between *G. hirsutum* and *G. barbadense*

Much of the genetic diversity of cotton can be quantified by the frequency of SNPs. In addition to 322,285 coding-region SNPs (cSNPs) and 173,334 intronic-region SNPs involved in 56,401 predicted genes [21], the majority (93.8%) of the 7,993,856 common SNPs were located in intergenic regions (Additional file 1: Figure S5). The allele frequency distributions of 44,250 nearly fixed cSNPs were highly diverged between *G. hirsutum* and *G. barbadense*. The number of nearly fixed cSNPs detected between 33 race accessions and 52 cultivars in *G. hirsutum* was 1179 (Additional file 1: Figure S6). The sequence divergence at the evolution level among accessions was further evaluated using the ratio of nonsynonymous (*Ka*) SNPs against synonymous (*Ks*) SNPs. The average *Ka*/*Ks* ratio was 0.49 for all common cSNPs. However, for 561 genes with nucleotide-binding site leucine-rich repeat domains, the ratio (0.73) was relatively higher, suggesting these genes are evolving more rapidly in response to co-evolving pathogens. The *Ka*/*Ks* ratios for the nearly fixed cSNPs were 0.57 between *G. hirsutum* and *G. barbadense*, and 0.91 between races and modern cultivars of *G. hirsutum*, indicating the existence of higher selection pressure during upland cotton domestication from wild to dooryard types and then field production.

We also identified 5784 protein-coding genes with premature stop codons or frameshifts resulting from 6661 SNPs and 2047 indels. A frameshift mutation occurred in a total of 1447 protein-coding genes resulting from 2047 indels (Additional file 9: Table S7). Of these, we found a flowering-related gene, Gh_D02G1411, homologous to *ABA OVERLY SENSITIVE 4* (*AtABO4*, AT1G08260) in *Arabidopsis*. The *abo4-1* plants were early flowering with lower expression of FLOWER LOCUS C and higher expression of FLOWER LOCUS T and changed histone modifications in these two loci [25]. Another interesting indel-containing gene encoding a cell wall-loosening protein, expansin A8 (*EXPA8*), played an important role in determining the rate and temporal period of fiber elongation and further fiber quality improvement [26].

**Fig. 2:**
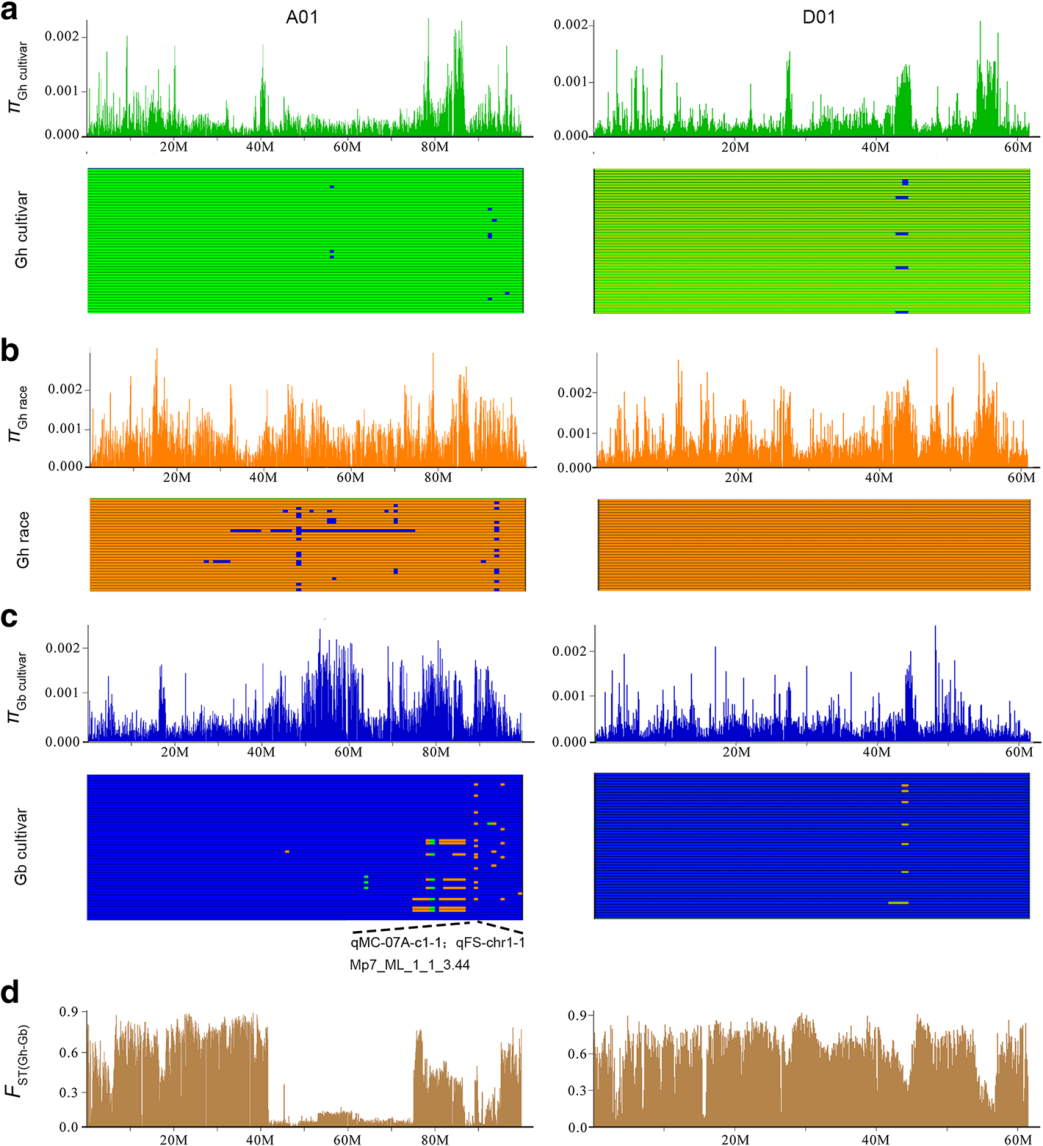
Characterization of the genetic diversity and introgression on chromosomes A01 and D01 in cotton. The levels of genetic diversity in *G. hirsutum* cultivars (*π*_Gh cultivar_) (**a**) and races (*π*_Gh race_) (**b**), the level of genetic diversity in *G. barbadense* (*π*_Gb cultivar_) (**c**), and the level of genetic differentiation between *G. hirsutum* and *G. barbadense* (**d**). For introgression analysis, the genetic backgrounds of *G. hirsutum* cultivars, *G. hirsutum* races, and *G. barbadense* cultivars are illustrated in *green* (**a**), *orange* (**b**) and *blue* (**c**), respectively

We examined the genetic diversity across the 26 chromosomes (Additional file 10: Table S8), and a strong signal of differentiation was observed at the whole genome level between *G. hirsutum* and *G. barbadense* accessions (Fig. 2 chromosomes A01 and D01 displayed as examples and Additional file 1: Figure S7). The fixation index values (*F*_ST_) were 0.63 and 0.65 in the A and D subgenomes, respectively, which were slightly higher than that between *indica* and *japonica* rice subspecies (*F*_ST_ = 0.55) [27] and much higher than that between *G. hirsutum* races and cultivars (*F*_ST_ = 0.10 for both subgenomes).

**Fig. 3:**
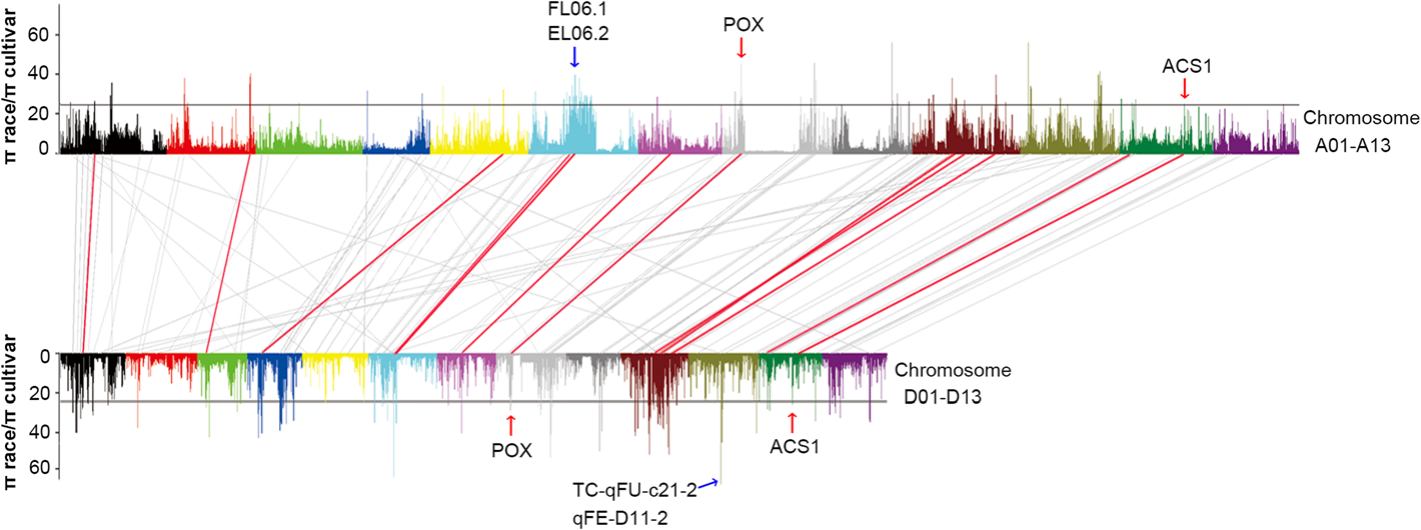
Identification and comparative analysis of the selective sweeps in *G. hirsutum*. The values of *π*_race_/*π*_cultivar_ were plotted against the position on each of the 26 chromosomes. The relationships between each selective sweep and its corresponding homologous region in the allotetraploid genome are indicated by *grey lines*. The 12 selective sweep pairs with high or modest selection signals in homoeologous regions are indicated by *red lines*. The *blue arrow* indicates the fiber quality related QTLs around the strongest selection signal locus in D11 and the longest selection region in A06. The *red arrow* indicates the POX and ACS1 genes in the A08/D08 and A12/D12 homoeologous regions

Whole-genome analysis identified 109 selective sweeps that spanned 3.4% of the *G. hirsutum* cotton genome through the comparison of 33 accessions of seven races and 52 modern cultivars (π_race_/π_cultivar_ > 25; Fig. 3; Additional file 11: Table S9). We investigated the genomic variation of *G. barbadense* at the 109 selective sweep regions identified in *G. hirsutum*. Compared with the sequence diversity at the whole genome level, the *G. barbadense* population did not show a significant change at the 109 selective sweeps (*π*_sweep_ =0.00055 versus *π*_genome_ =0.00056), indicating different selection pressures on the *G. hirsutum* and *G. barbadense* genomes. These genomic data further support our previous view that the two species were domesticated independently [1, 8]. The phenomenon is similar to the dual domestication processes in common beans, where two divergent populations of *Phaseolus vulgaris* were independently domesticated in Mesoamerica and South America [28], as well as in cultivated rice, where *Oryza sativa* and *Oryza glaberrima* were independently domesticated in Asia and Africa [29].

*G. hirsutum* and *G. barbadense* had similar levels of sequence diversity. The nucleotide diversity levels of the A and D subgenomes were 0.00075 and 0.00073, respectively, in *G. hirsutum*, and 0.00061 and 0.00051, respectively, in *G. barbadense*. It is possible that these numbers have been underestimated because tetraploid cotton genomes have large proportions of repetitive sequences and paralogs [21] similar to those in other large-genome plants such as maize [30]. To provide an indication of the mapping resolution in genome-wide association studies, the decay rate of linkage disequilibrium (LD) was calculated. The average pairwise correlation coefficient (*r* ^*2*^) dropped from 0.6 at 1 kb to 0.3 at 1000 kb in *G. hirsutum*. This slow LD decay might have resulted from inbreeding nature in cotton. Moreover, as expected, a slower LD decay rate was found in cultivars than in the wild species and primitive races (Additional file 1: Figure S8).

### Asymmetric introgression between *G. hirsutum* and *G. barbadense*

In spite of the strong genetic divergence between *G. hirsutum* and *G. barbadense*, the interspecific hybrids of the two cultivated species are fertile and grow vigorously, and some F_1_ hybrids are commercially produced [31]. Cotton breeders have worked diligently to introduce some desired alleles from one species to another in order to increase genetic diversity. To analyze introgression between tetraploid cottons, a recently developed “3-population test” method [32, 33] was used for modeling. Among all possible scenarios, we found evidence of introgression events between *G. hirsutum* races and *G. barbadense* cultivars (f3 = −0.1223, Z score = −253.4; Additional file 12: Table S10). These introgression events were successfully traced using the population-scale genomic data generated in the present study (Additional file 1: Figure S9). On average, 0.2% genomic regions in 137 accessions (excluding the ten outgroup accessions) showed obvious introgression events (384 introgression events detected in at least two accessions) (Additional file 13: Table S11). Intriguingly, the introgression events were significantly biased towards the gene flow from *G. hirsutum* into *G. barbadense* than that from *G. barbadense* into *G. hirsutum* (265 versus 119, Fisher’s exact test, *P* = 8.04E-08; Fig. 2; Additional file 14: Dataset 2). Moreover, more introgression events were found in the A subgenome (250) than in the D subgenome (134) (Fisher’s exact test, *P* = 2.29E-05). A previous study described interspecies introgression in a limited population of 11 *G. hirsutum* and three *G. barbadense* [23]; however, the researchers used two diploid progenitor genomes [34, 35] instead of two published tetraploid genomes [21, 36] as the reference. Many structure variations have occurred after the formation of tetraploid cotton compared to two corresponding progenitors. From our previous colinearity analysis, the overall gene order and colinearity were largely conserved between our A and D subgenomes [21] and the D progenitor genome [34], but this colinearity was not obvious between our A and D subgenomes and the A progenitor genome [35], partly due to numerous examples of mis-assemblies in the A progenitor genome, as we reported before [21], and partly because *G. arboreum* is an important cultivated diploid species and may have undergone some of its own chromosomal rearrangements during its evolution and improvement. Additionally, a larger population in the present study will be helpful to identify the introgression event more comprehensively compared to a previous study that used limited samples [23].

Across the allotetraploid cotton genomes, we found 11 regions of extensive introgressions, with the greatest density in chromosome A1 (Fig. 2; Additional file 14: Dataset 2; Additional file 15: Table S12). Analysis of QTLs has provided genetic evidence that these regions were associated with fiber quality traits (Additional file 16: Table S13). We observed 169 introgression events from six primitive races of *G. hirsutum* into Sea Island cottons of the *G. barbadense* species, such as Coastland R4-4, Seabrook, and West of Andes, instead of Tanquis, whose fiber was medium staple (23.8 to 27.0 mm in length) and was coarse. This fiber performance of the landraces such as Tanquis is typified by current cottons of Peru, where the ancestral *G. barbadense* originated [9]. Genomic evidence from the present study reveals subsequent introgressions from the local wild *G. hirsutum* or races into *G. barbadense* during its movement northward through inland Mesoamerica, from the Yucatan peninsula to the Caribbean Islands, where Sea Island cotton originally formed and was then introduced to the coastal states of the southeastern United States (Additional file 1: Figure S10). No introgression evens occurred from *richmondi* to Sea Island cottons, probably because of restricted geographical positions along the Pacific side of the Isthmus of Tehuantepec or limited collected accessions.

Among these 169 introgression events from *G. hirsutum* races into *G. barbadense* accessions, four events observed in Giza36, Giza80, Pima S-1, and Pima S-2 were detected in the same introgression region, the ChrA10.57.block (Additional file 14: Dataset 2). This block overlaps a QTL for fiber length (qFL-A10-2) [37]. In this block, we annotated 11 genes, of which five were potentially related to seed and fiber development, mainly involved in auxin transport (auxin efflux carrier gene) [38], transcription factors (WD40 repeat-like superfamily genes) [39, 40], and carbohydrate metabolism (o-fucosyltransferase gene, sucrose phosphate synthase gene, and beta galactosidase) [41] (Additional file 17: Table S14). In the ChrA11.88.block, which is also an introgression region from *G. hirsutum* races into four central Asia type *G. barbadense* accessions (CCCP1243, XH 3, XH 11, and XH 29), at least nine of 27 genes are potentially related to disease resistance, including two TIR-NBS-LRR genes [42], five pectin methylesterase inhibitor genes [43], and two dirigent-like protein genes [44] (Additional file 14: Dataset 2; Additional file 17: Table S14). We found 1061 genes in 169 introgression events from *G. hirsutum* races to *G. barbadense* and 665 genes in 96 events from *G. hirsutum* cultivars to *G. barbadense*. Interestingly, the genes in the former were enriched in developmental processes, such as reproduction, epithelial cell development, and cell proliferation, possibly allowing the allopolyploid to survive and even thrive considering its wide adaption. In contrast, the latter genes were enriched in cellular homeostasis, fatty acid oxidation, and lipid catabolic processes (Additional file 18: Table S15). In the 119 introgression events from *G. barbadense* to *G. hirsutum*, we further found 587 genes enriched in lipid metabolic and carbohydrate metabolic processes (Additional file 18: Table S15). These results support the idea that such introgressions confer beneficial traits such as fiber quality and photoperiod neutrality and are responsible for the creation of the Sea Island cotton germplasm, as reported previously [5, 9, 12, 20, 31].

In spite of a low introgression rate, some *G. barbadense* segments were found to be introgressed into *G. hirsutum* races (Additional file 14: Dataset 2). These interspecific gene flows might have occurred during the northward movement of *G. barbadense* (Additional file 1: Figure S10).

Modern Egyptian-type ELS cultivars showed genomic signatures of *G. hirsutum* race introgressions in chromosome A1 (81–84 Mb, 88–89 Mb), A10 (21–22 Mb, 56–57 Mb), and D11 (10–11 Mb); the American-Pima type in A1 (77–78 Mb, 84–89 Mb) and A10 (56–57 Mb); and the Central Asia type in D1 (42–44 Mb), D9 (3–4 Mb, 5–6 Mb, 49–50 Mb), D10 (6–7 Mb, 57–62 Mb), and D11 (11–16 Mb, 63–64 Mb) (Additional file 13: Table S11; Additional file 14: Dataset 2), suggesting a distinct improvement in the Central Asian type ELS cultivars. Some introgression events, such as those in chromosome A1, were previously reported using restriction fragment length polymorphism markers [20], in which the *G. hirsutum* allele was found in 48 (94%) of the 51 *G. barbadense* collections, including Egyptian and Pima cottons. Furthermore, modern breeding has enhanced gene flow and post-domestication introgressions through deliberate hybridization between these two species. For example, targeted introgressions from *G. barbadense* cultivars have been used to develop Acala cultivars, which improved upland cotton’s fiber quality and *Verticillium* resistance [45].

### Signatures of selection and adaptive trait associations in *G. hirsutum*

The genetic diversity in modern cultivars was found to be low (*π*_cultivar_ = 0.00074)—only 34.2% (32.4 and 35.0% for the A and D subgenomes, respectively) of that in races (*π*_race_ = 0.00216)—indicating a strong genetic bottleneck during upland cotton domestication. This diversity level is close to that in *japonica* rice (33%) [27] and much lower than that in maize (83%) [46] and *indica* rice (75%) [27].

Phylogenetic analysis of the 109 selective sweeps revealed a strong selection pressure in nearly all cultivars of *G. hirsutum*. The average selection signal (*π*_race_/*π*_cultivar_ = 32.8) in the A subgenome was close to that in the D subgenome (*π*_race_/*π*_cultivar_ = 35.0), but the sweep regions between the A and D subgenomes were largely different. These selective sweeps domesticated for fiber yield and fiber qualities provide a resource for molecular breeding of *G. barbadens*e in the future.

Interestingly, 12 homoeologous pairs of selective sweeps with high or modest selection signals (π_race_/π_cultivar_ ranging from 15.4 to 39.6) were detected between the A and D subgenomes (Fig. 3), probably due to selection of a common set of domestication genes. For example, peroxidase genes (*POX*, Gh_A08G0711/Gh_D08G0829) and ACC synthase genes (*ACS1*, Gh_A12G0969/Gh_D12G1017) participating in ethylene biosynthesis were co-selected within the overlapped regions of the selective sweeps of the A08/D08 and A12/D12 homoeologous pairs, and these genes play key roles in fiber elongation [47, 48].

To investigate the contribution of selective sweeps in the domestication for fiber yield and fiber qualities in *G. hirsutum*, the overlap between selective sweeps and QTLs of various agronomic traits was further examined. A total of 211 fiber quality- and lint yield-related QTLs were around 67 selective sweeps (Additional file 19: Table S16). The locus associated with the strongest selection signal (*π*_race_/*π*_cultivar_ = 100.0) was located on chromosome D11 and overlapped with several QTLs controlling fiber length (Fig. 3; Additional file 19: Table S16). Another strong selective sweep was located on chromosome A6, covering a very long genomic interval (21.6 Mb) that overlapped QTLs for fiber length and lint percentage (Additional file 19: Table S16). Fiber length and lint yield have greatly increased during domestication from wild type, primitive races, and advanced types to modern cultivars.

The examination of gene expression in selective sweeps responsible for various agronomic trait QTLs indicated some casual genes may be related to this domestication. Of the 1058 genes identified in all 109 selective sweeps, 723 were expressed in fiber development stages. Additionally, 236 of these 723 genes had significantly higher expression levels during fiber development in domesticated cotton (TM-1) than those in two wild relatives (TX665, *G. hirsutum* var. *palmeri* and TX2094, G. *hirsutum* var. *yucatanense*) (Additional file 20: Table S17).

**Fig. 4:**
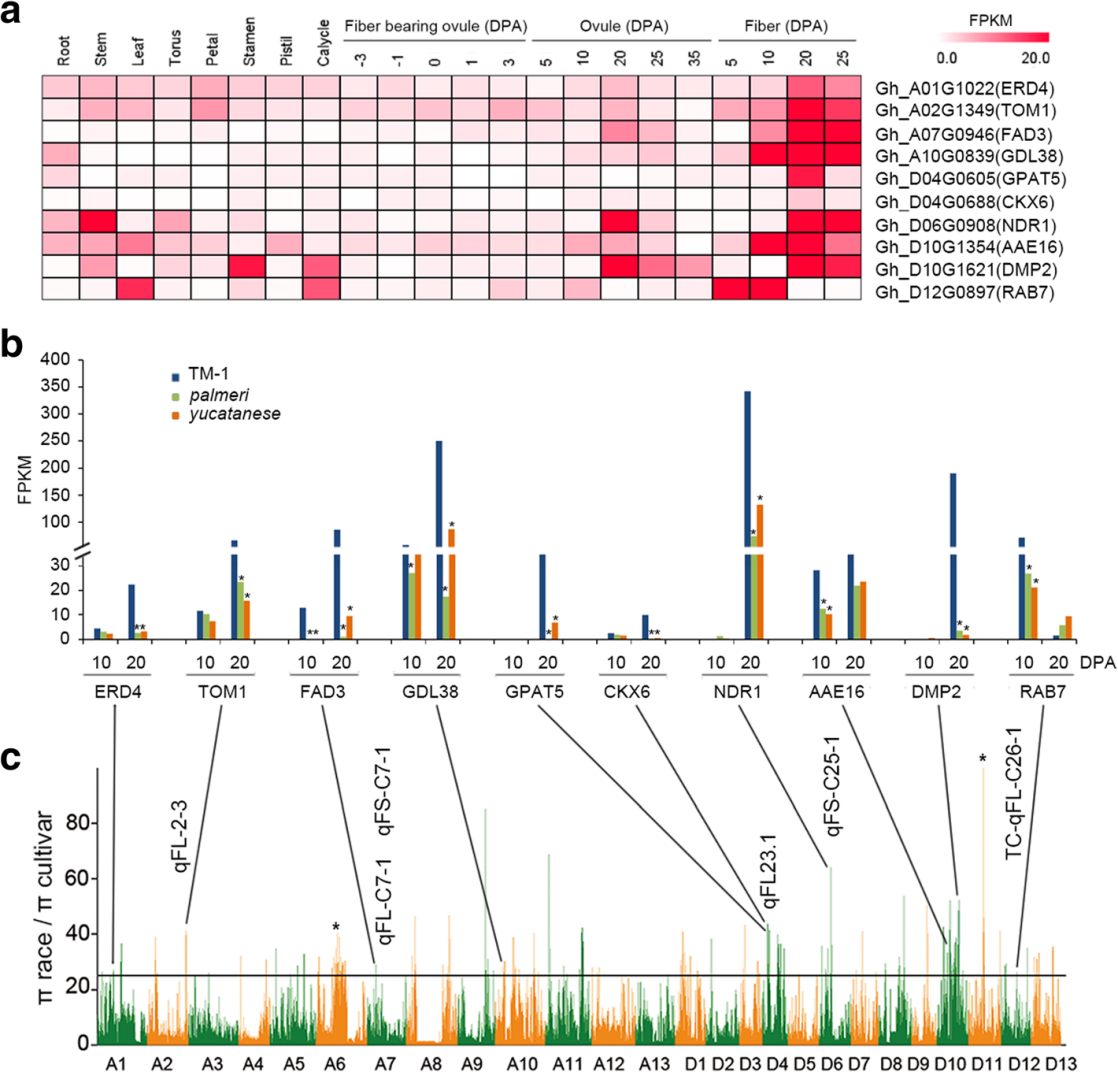
Expression pattern of ten fiber-related genes related to cotton fiber quality. **a** Expression pattern of ten genes related to fiber quality in distinct tissues. **b** Expression level of these genes in domesticated cotton (TM-1) and two wild relatives (TX665, *G. hirsutum* var. *palmeri*; TX2094, G. *hirsutum* var. *yucatanense*). Fisher’s exact test, ^*^ *P* value <0.05 and fold change >2. **c** Identification of 109 selective sweeps through comparisons of races and cultivars in *G. hirsutum*. The values of *π*_race_/*π*_cultivar_ were plotted against the position on each of the 26 chromosomes. The *horizontal line* indicates the genome-wide threshold of selection signals (*π*_race_/*π*_cultivar_ >25). *Asterisks* indicate the strongest selection signal locus in D11 and the longest selective sweep in A06. Lines linking **b** and **c** indicate the gene locus in selection sweeps. The fiber quality-related QTLs around these gene loci are shown beside these lines

Using RNA-seq data from multiple tissues, we found that the proportions of genes that were expressed during fiber development and seed germination were higher in the selective sweeps than in the whole genome (Additional file 1: Figure S11). Within the selective sweeps, 76 fiber- and 115 seed germination-related genes (Additional file 21: Table S18; Additional file 22: Table S19; Additional file 23: Table S20) were identified based on their expression profiles. Ten of these 76 genes were expressed at significantly higher level in TM-1 than in palmeri and yucatanense races (Fig. 4). For instance, a cytokinin oxidase gene (*CKX6*, *Gh*_*D04G0688*) was associated with increased fiber and seed yield [49]; a fatty acid desaturase (*FAD3*, Gh_07G0946) was required for the specific membrane structure of fiber cells and genes encoding very long chain fatty acid (*VLCFA*) synthase for fiber cell elongation [47, 48]. These results suggest potential roles in the improved fiber qualities of domesticated cotton.

Of the 115 seed germination-related genes, Gene Ontology analysis showed an enrichment for genes involved in biological processes related to histone methylation and ethylene signaling pathways, which are required for the positive regulation of seed dormancy [50] (Additional file 23: Table S20). For instance, the gene encoding an AP2/ethylene response factor (ERF1, Gh_D10G1537) was found in the selective sweeps. The loss-of-function *ap2* mutant showed increased seed mass relative to the wild type in *Arabidopsis* [51]. Overexpression of *OsERF1* in *Arabidopsis* up-regulated the expression of two known ethylene-responsive genes, leading to short hypocotyls/roots and the production of fewer seeds or no siliques at all [52]. Another gene, Gh_A10G0771, was homologous to a RING E3 ubiquitin ligase in *Arabidopsis*, which regulated the stability of the cyclin-dependent kinase inhibitor KRP1 and further negatively regulated the cell number and seed size [53, 54]. KRP1 is the target of the ubiquitin-proteasome pathway recently found to play an important part in plant seed size determination [55, 56]. However, the molecular mechanisms of antagonistic function in the complex regulation of seed dormancy are still unclear. The candidate genes identified in the selective sweeps are valuable for future functional analyses of seed dormancy reduction during domestication.

## Conclusions

Resequencing and genome-wide analysis of diverse *G. hirsutum* and *G. barbadense* wild accessions and modern cultivars have provided a comprehensive genome-wide assessment of a fiber crop and enabled us to better understand the evolution, diversity, and domestication of allotetraploid cottons. Strong genomic divergence between *G. hirsutum* and *G. barbadense* led to dual domestication events of these two cultivated species, while reciprocal, but asymmetric, introgression between them has greatly improved their productivity and fiber quality. Although both are commonly grown as fiber crops, they have been domesticated or improved toward different breeding goals: *G. hirsutum* for its high yield and wide adaptation, and *G. barbadense* for its superior fiber quality. This large amount of new genomic resources will substantially improve genetic mapping, gene identification, and molecular breeding in cotton. Specifically, under the guidance of sequence information, the favorable alleles that are associated with high yield potential and wide adaptation in *G. hirsutum* and with fiber quality in *G. barbadense* can be introgressed between the gene pools to further improve cotton production.

## Methods

### Sampling

In order to represent the rich genetic diversity and wide geographical distribution of cotton, we selected seven geographical races of *G. hirsutum* (“marie-galante’, “punctatum”, “richmond”, “morrilli”, “palmeri”, “latifolium”, and “yucatanense”) [11], a variety of *G. hirsutum* cultivars, including four major types—Acala, Delta, Plains, and Eastern—from the USA, and other domesticated subtypes from Brazil, India, Africa, and China. Furthermore, *G. barbadense* cultivars, including American Pima, Egyptian, Peruvian Tanquis, and other subtypes from Russia and China, were also sampled. Although extant wild *G. barbadense* populations have been reported in Guayas and Los Rios in Ecuador and Tumbes in Peru [31, 57], the search for truly wild accessions is complicated since the wild-to-domesticated continuum in *G. barbadense* does not have obvious categorical distinctions. Another three wild allotetraploid species, *G. darwinii*, native to the Galapagos Islands, *G. tomentosum* from the Hawaiian Islands, and *G. mustelinum*, an uncommon species restricted to a relatively small region of northeast Brazil, as well as *Thespesia populneoides* (Roxb.) Kostelas, a species in the mallow family (Malvaceae) closely related to the cotton genus (*Gossypium*), were chosen to form an outgroup. Detailed information on the 147 cotton accessions is listed in Additional file 2: Table S1.

### Library construction and sequencing

For each cotton accession, young leaf tissues from a single plant were collected for genomic DNA extraction using a standard cetyl trimethylammonium bromide (CTAB) protocol [58]. Paired-end sequencing libraries with insert sizes ranging from 300 to 500 bp were constructed according to the manufacturer’s instructions (Illumina, San Diego, CA, USA). All libraries were sequenced on the Illumina HiSeq 2000 platform. A total of 1.8 terabases of genomic sequence data was also generated with an average 5× genome coverage for each cotton accession.

### Genotype calling and SNP identification

All sequence reads were aligned against the reference genome sequence (*G. hirsutum* cv. TM-1) [21] using Smalt software (version 0.57, http://www.sanger.ac.uk/resources/software/smalt/). The parameter for the read mapping was “smalt_x86_64 map -i 700 -j 50 -m 60”. For the oriented 1.9-Gb genome sequence, 36.2% of reads were mapped to the A subgenome and 23.9% to the D subgenome for *G. hirsutum*. Additionally, 10.5% of the total reads were mapped to the A subgenome scaffold (326.3 Mb); 1.9% of the total reads were mapped to the D subgenome scaffold (61.5 Mb); 4.1% of the total reads were mapped to unclassified scaffold (124.6 Mb); and 23.4% of the total reads had no unique location in the mapping process. Only reads with a unique mapping position in the oriented reference genome and a mapping score higher than 60 were used. If reads had equal matching scores in the A and D subgenomes, the reads were excluded from the SNP calling procedure. The software package Ssaha pileup (http://www.sanger.ac.uk/resources/software/ssaha2/) was used to find candidate SNPs that required support from at least two sequence reads. Only the non-singleton SNPs, defined as those where more than two accessions demonstrate the presence of the alternative alleles, were retained. We then filtered the polymorphic sites with minor allele frequency (5%) and missing rate (10%). We randomly removed the polymorphic sites in the high homoeologous polymorphic sites until the remaining polymorphic sites were at least 10 bp away from neighboring polymorphic sites and got the final polymorphic datasets. In the filtering process, we found that about 40% of non-singleton polymorphic sites had missing rates of less than 10%. We required that the common SNPs had a minor allele frequency (MAF) greater than 5% and a missing data rate less than 10%. We only analyzed the SNPs that were located in the 26 pseudomolecules of the TM-1 assembly, and the SNPs in the small scaffolds were removed. The SNPs were annotated using the GFF files (the annotation file of all coding regions of each gene) of the TM-1 reference genome sequence. The software KaKs_Calculator was then used to compute the Ka/Ks ratio.

### Indel identification and annotation

Pindel software (version 0.20) [59] was used to identify the indels from the sequence reads. In order to identify the indels, we used the Smalt outputs and kept only three kinds of reads: (1) the paired-end sequence reads that had a unique match on one side and no match on the other side; (2) the paired-end reads where small indels were detected in the Smalt output file; and (3) the paired-end reads that had a unique match in the genome but a low alignment score. We converted the filtered reads into the Pindel input file format. Only the indels that had the support of more than three reads and were detected in at least two accessions were retained as candidate indels. The genomic position of each indel was checked against the GFF file to allow for cotton genome annotation. Genes with indels causing open reading frame changes were considered to have a mutation with a large effect.

### SNP validation

We used two methods to validate the SNP calling. First, we used the assemblies of the TM-1 and XH21 genomes to identify the genotypes of TM-1 and XH21 at the SNP site, respectively. We compared the genotypes in the assembled sequences against those in the SNP datasets called from the resequencing data and calculated the SNP accuracy rate. Second, we further randomly selected 68 SNPs and carried out PCR-based sequencing in 11 randomly selected accessions (seven *G. hirsutum* and four *G. barbadense* accessions) with three replicates. We aligned all the PCR products against the TM-1 genome using BLAST (version 2.2.28), and the reads with mapping lengths >90% and identity >80% were used for SNP validation. Using the alignment results, we retrieved the genotypes in 11 accessions for each SNP site. Only the genotypes consistent across three replicates were used to calculate the accuracy (Additional file 24: Table S21; Additional file 25: Table S22).

### Population structure analysis

Using the Ssaha pileup package, we generated an SNP matrix for 147 cotton accessions and calculated the simple matching coefficient of whole-genome SNPs as the genetic distance. We used Phylip software (version 3.69) [60] to generate the neighbor-joining tree. Dendroscope [61] was used to display the phylogenetic tree. The missing data in the cotton SNP genotype dataset were imputed using Beagle (version 3.3.2) [62]. We converted the raw genotype matrix to the Beagle unphased format and imputed using the parameter “-unphased”. We performed population structure analysis using STRUCTURE [63] and principal component analysis was performed using EIGENSTRAT software [64].

### Population genetics analysis

According to the phylogenetic tree, the 147 cotton accessions could be divided into four major groups: *G. hirsutum* races, *G. hirsutum* cultivars, *G. barbadense* cultivars, and the outgroup. For each group, we calculated the level of genetic diversity (*π*) in each 100-kb interval across the cotton genome. The level of population differentiation, *F*_ST_, was calculated in 100-kb intervals. During the identification of selective sweeps in *G. hirsutum* cultivars, we measured the level of genetic diversity in races and in cultivars. We used the ratio π_race_/π_cultivar_ to evaluate selection signals. Genomic regions with diversity values lower than 0.001 in both races and cultivars were removed from the ratio calculation. Permutation tests were performed to estimate the false positive rate of the selection sweeps in order to validate the accuracy of the diversity ratio, with a ratio of 25 used as the threshold [65]. LD was calculated using Haploview software, with the default settings [66]. To model the mixture between different populations, the software TreeMix was used to perform a “3-population test” [32, 33]. In the test, the f3 statistic is the normalized product of the frequency difference. If there is no mixture, the expected value of the f3 statistic is positive.

### Introgression between *G. hirsutum* and *G. barbadense*

We screened 26 cotton chromosomes to detect the genomic regions of genetic introgression. A recently developed “3-population test” method [32, 33] was used for modeling the introgression between tetraploid cottons. Moreover, we divided the whole cotton genome into 1-Mb blocks and calculated the genetic distances through simple matching coefficients between the 147 cotton accessions. Within the introgression regions, we detected a total of 384 introgression events that were observed in at least two accessions. Phylip software (version 3.69) was used to generate the NJ tree, and the R software package “ape” was used to display the tree file. We checked each phylogenetic tree and searched for *G. hirsutum* accessions located within the *G. barbadense* clade or *G. barbadense* accessions located within the *G. hirsutum* clade.

### Calculation of gene expression level

RNA-Seq data from distinct tissues have been reported in previous TM-1 genome sequencing research [21]. The raw transcriptome data of two wild cotton relatives (TX665, *G. hirsutum* var. *palmeri*; TX2094, G. *hirsutum* var. *yucatanense*) were downloaded from the Sequence Read Archive (http://www.ncbi.nlm.nih.gov/sra/SRX202873) [34]. We calculated the expression of each gene using fragments per kilobase of exon model per million mapped reads (FPKM) with Cufflinks (version 2.1.1) [67]. Gene expression in different tissues is listed in Additional file 21: Table S18. Genes that had different transcript levels among these 14 tissues were defined at *P* < 0.05 using a Student’s *t*-test. If the gene expression in one tissue did not represent a 95% confidence level of the T distribution of the other 13 tissues, it was identified as a special tissue-related gene.

## Additional files

Additional file 1: Supplementary Figures S1–S11. (DOCX 4378 kb)
Additional file 2: Table S1. Summary of the 147 cotton samples and their sequencing data. (XLSX 28 kb)
Additional file 3: Table S2. The 68 SNPs validated in 11 randomly selected accessions by PCR methods. (XLSX 20 kb)
Additional file 4: Table S3. SNP accuracy verified using PCR-based sequencing. (XLSX 10 kb)
Additional file 5: Table S4. Summary of detected indels based on the genome resequencing data. (XLSX 6692 kb)
Additional file 6: Table S5. Summary of detected SVs based on the genome resequencing data. (XLSX 837 kb)
Additional file :7 Table S6. Distribution of SNPs and indels in 26 chromosomes of TM-1. (XLSX 11 kb)
Additional file 8: Dataset 1. A phylogenetic tree with full accession names. (PDF 180 kb)
Additional file 9: Table S7. Summary information of 1447 indel-containing genes. (XLSX 532 kb)
Additional file 10: Table S8. Levels of genetic differentiation in different chromosomes. (XLSX 11 kb)
Additional file 11: Table S9. Genome-wide detection of selective sweep regions in upland cotton domestication. (XLSX 25 kb)
Additional file 12: Table S10. The results of the 3-population test. (XLSX 11 kb)
Additional file 13: Table S11. Genomic regions with introgressions between *G. barbadense* and *G. hirsutum. (XLSX 25 kb)*

Additional file 14: Dataset 2. Whole-genome analysis of genetic introgressions in allotetraploid cotton. (PDF 111 kb)
Additional file 15: Table S12. Genomic regions with extensive introgressions between *G. barbadense* and *G. hirsutum. (XLSX 12 kb)*

Additional file 16: Table S13. Cotton QTLs that overlapped with the introgression regions. (XLS 81 kb)
Additional file 17: Table S14. Candidate genes in two introgression regions from *G. hirsutum* race to *G. barbadense*. (XLSX 10 kb)
Additional file 18: Table S15. Functional enrichment of genes involved in introgression events between *G. hirsutum* and *G. barbadense*. (XLSX 11 kb)
Additional file 19: Table S16. List of fiber quality- and lint yield-related QTLs overlapping selected sweeps. (XLSX 34 kb)
Additional file 20: Table S17. Differently expressed selective sweep related genes between domesticated cotton and wild relatives. (XLSX 26 kb)
Additional file 21: Table S18. Gene expression level of 1058 genes identified in 109 selection sweeps. (XLSX 345 kb)
Additional file 22: Table S19. List of genes related to cotton fiber development that overlapped with the selective sweeps in *G. hirsutum*. (XLSX 14 kb)
Additional file 23: Table S20. List of genes related to cotton seed germination that overlapped with the selective sweeps in *G. hirsutum*. (XLSX 17 kb)
Additional file 24: Table S21. Primers for SNP accuracy validation. (XLSX 12 kb)
Additional file 25: Table S22. The position of 68 SNPs located in the 24 regions amplified by PCR. (XLSX 12 kb)
