# Supplementary material for: Genomic insights into divergence and dual domestication of cultivated allotetraploid cottons
Source: Genome Biol. 2017 Feb 20;18:33. doi: 10.1186/s13059-017-1167-5 (PMC5317056; doi:10.1186/s13059-017-1167-5)
Supplement: Additional file 1: — Supplementary Figures S1–S11. (DOCX 4378 kb) [file 13059_2017_1167_MOESM1_ESM.docx]

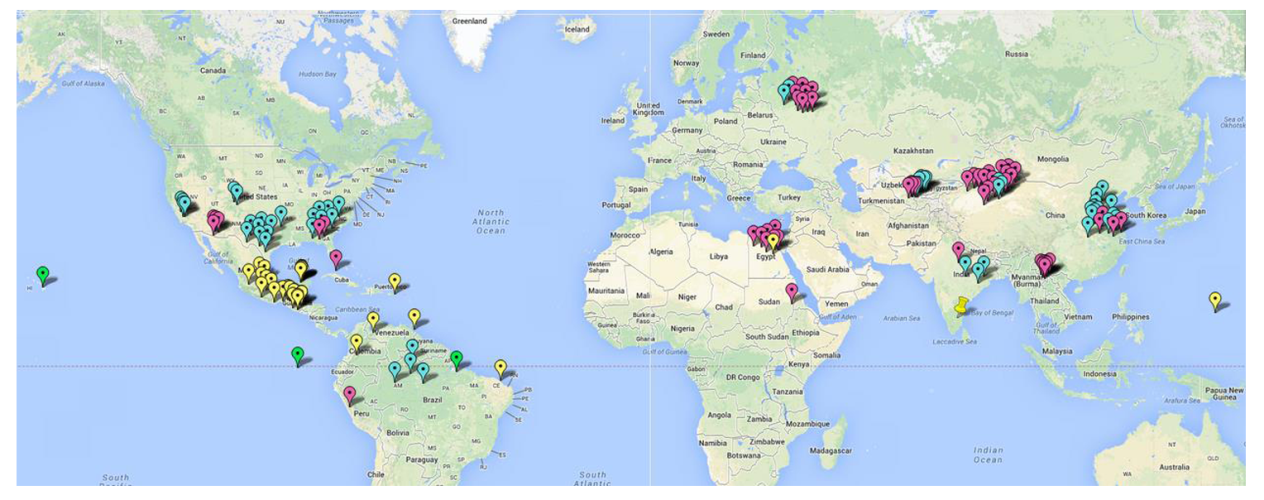


**Additional file 1: Figure S1. Geographic distribution of the diverse allotetraploid cottons.** We selected 33 primitive landraces (yellow), 52 cultivars (light blue) in *G. hirsutum*, and 57 accessions in *G. barbadense* (pink) from across the world. Furthermore, we included three wild allotetraploid species, *G. tomentosum* (AD)_3_, *G. mustelinum* (AD)_4_ and *G. darwinii* (AD)_5_ (green), for an interspecific comparison at the polyploidy level, as well as *Thespesia populneoides* (Roxb.) Kostelas (yellow thumbtack), as an outgroup. This map was produced with Google Maps.

**
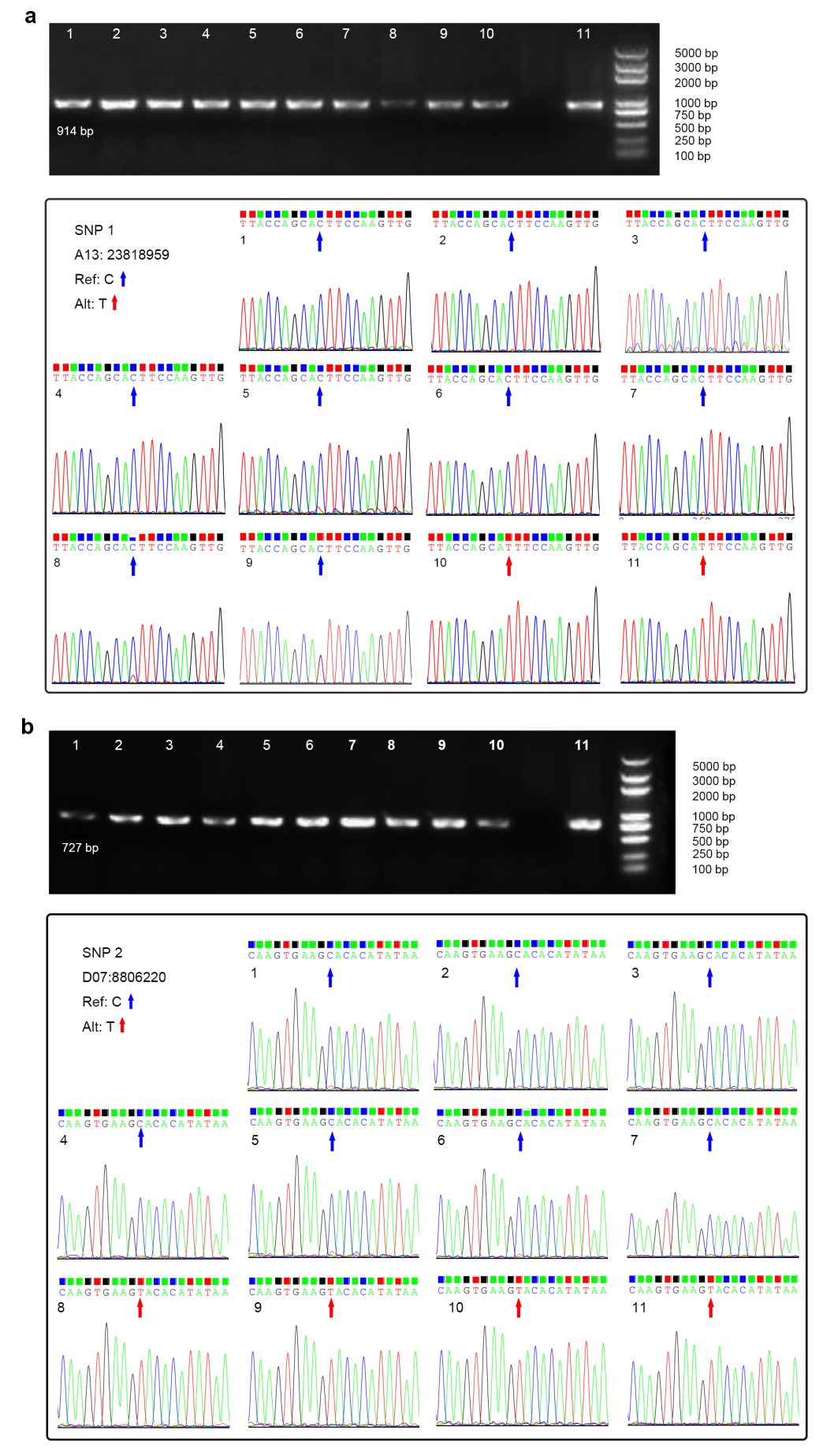
**

**Additional file 1: Figure S2. SNP accuracy validation.**  Two SNPs at Chromosome A13: 23819030 and D07: 8806220 were validated in 11 materials using PCR method with three duplications. The amplification products were 914 and 727bp length, respectively. For the SNP site, blue arrows indicate the reference type and red arrows indicate the altered type.


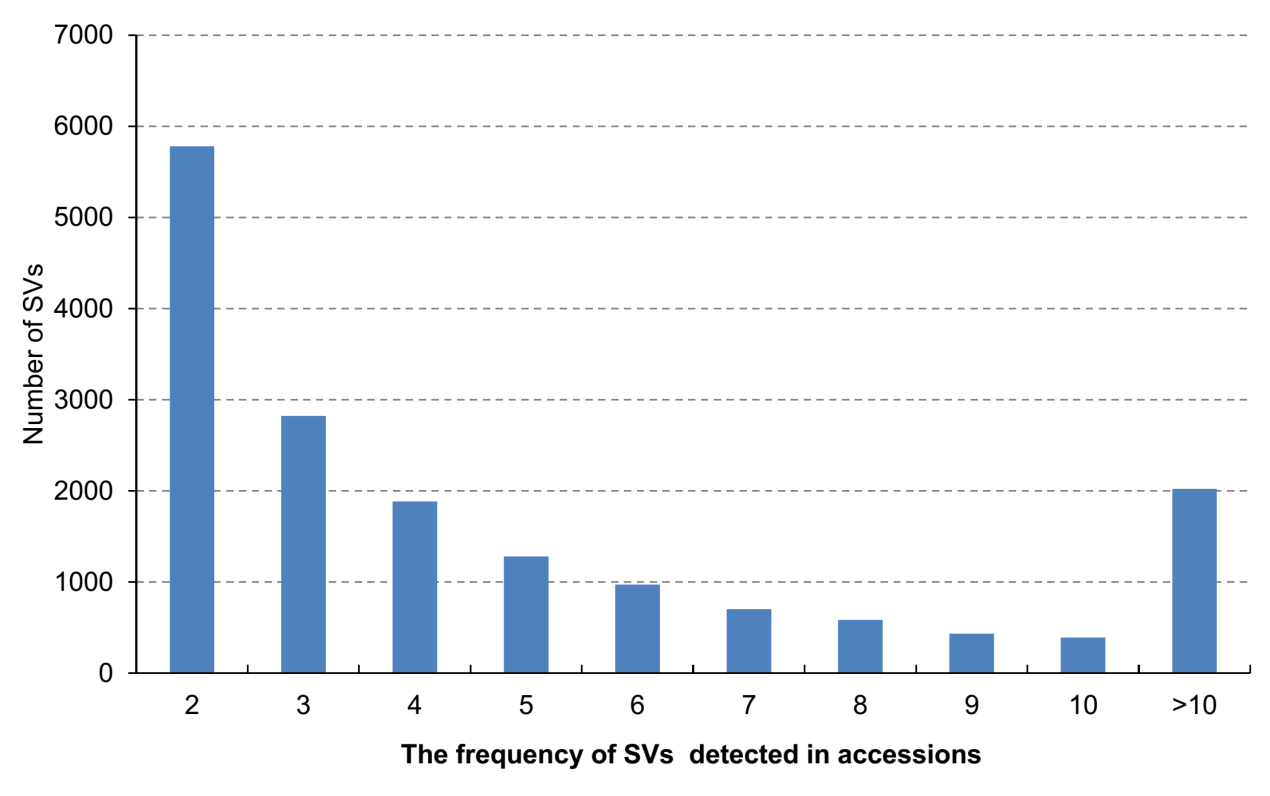


**Additional file 1: Figure S3. The frequency of SVs detected in accessions.** All these SVs were detected at least in two accessions. The X axis indicated the number of accessions in which the same SVs were detected. The Y axis indicated the corresponding SVs number on different frequency.


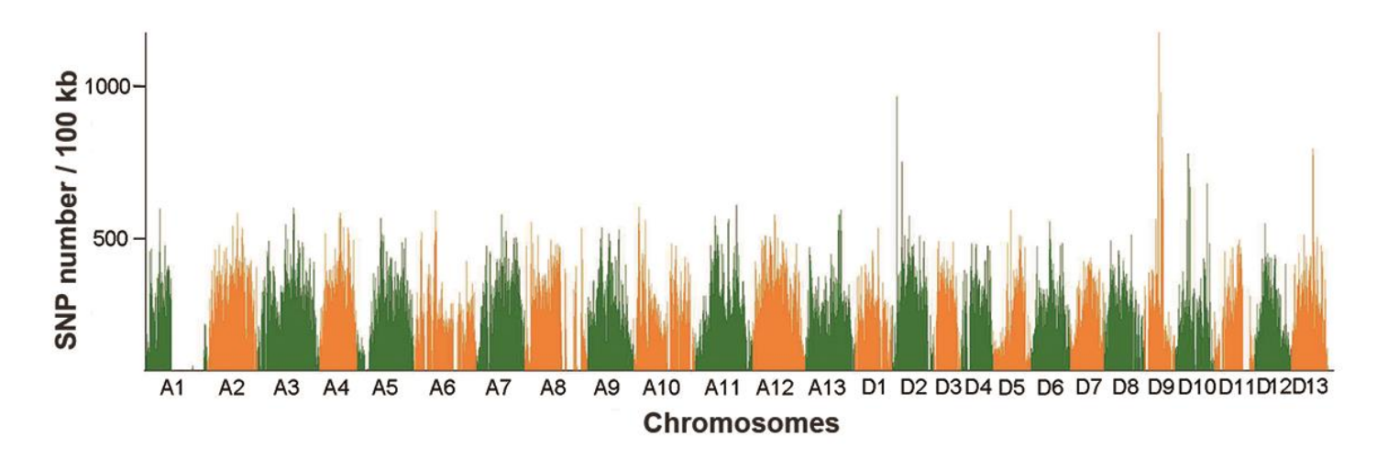


**Additional file 1: Figure S4. Distribution of the nearly-fixed SNPs between *G. hirsutum* and *G. barbadense*.** Totally 2,752,128 SNPs have an allele frequency of >95% in *G. hirsutum* or *G. barbadense* and <5% in the other species. The densities of the highly differentiated SNPs are plotted against position on each of 26 cotton chromosomes.


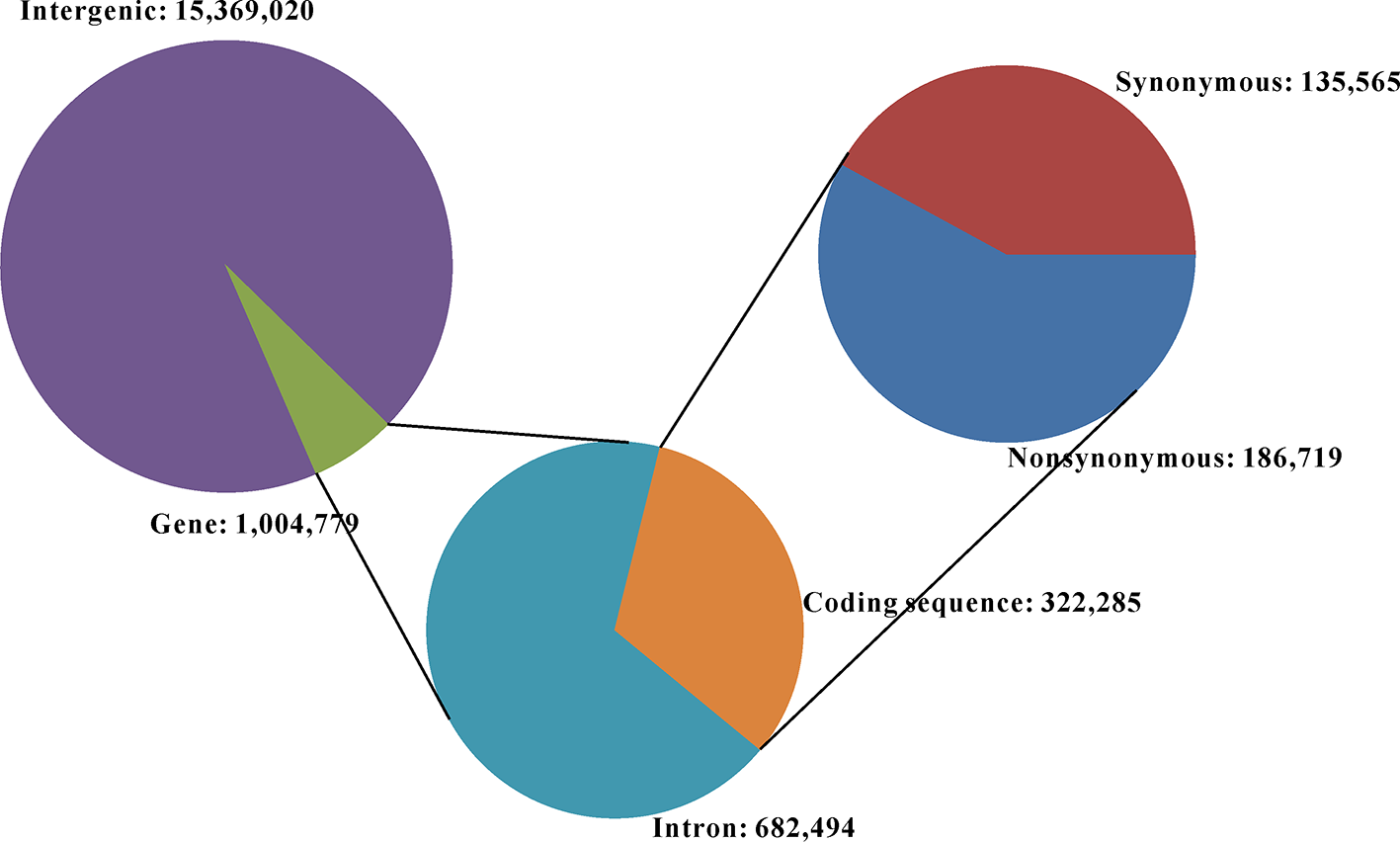


**Additional file 1: Figure S5. The distribution of the SNP patterns in the cotton genome.** The three circles represent the distribution of SNPs in genic and intergenic regions, the distribution of SNPs in exonic and intronic regions, and the cSNPs with synonymous and non-synonymous effects.


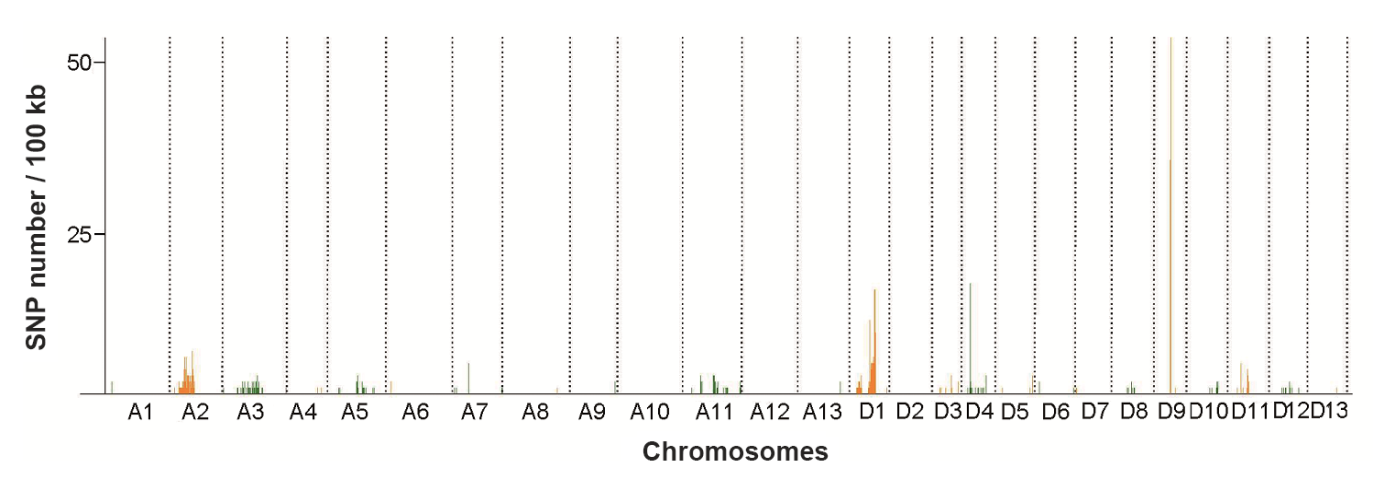


**Additional file 1: Figure S6. The distribution of the nearly-fixed SNPs between the races and cultivars in *G. hirsutum*.** Totally 1,179 highly differentiated SNPs between races and modern cultivars were identified in *G. hirsutum*. The densities of the highly differentiated SNPs are plotted against position on each of 26 cotton chromosomes.


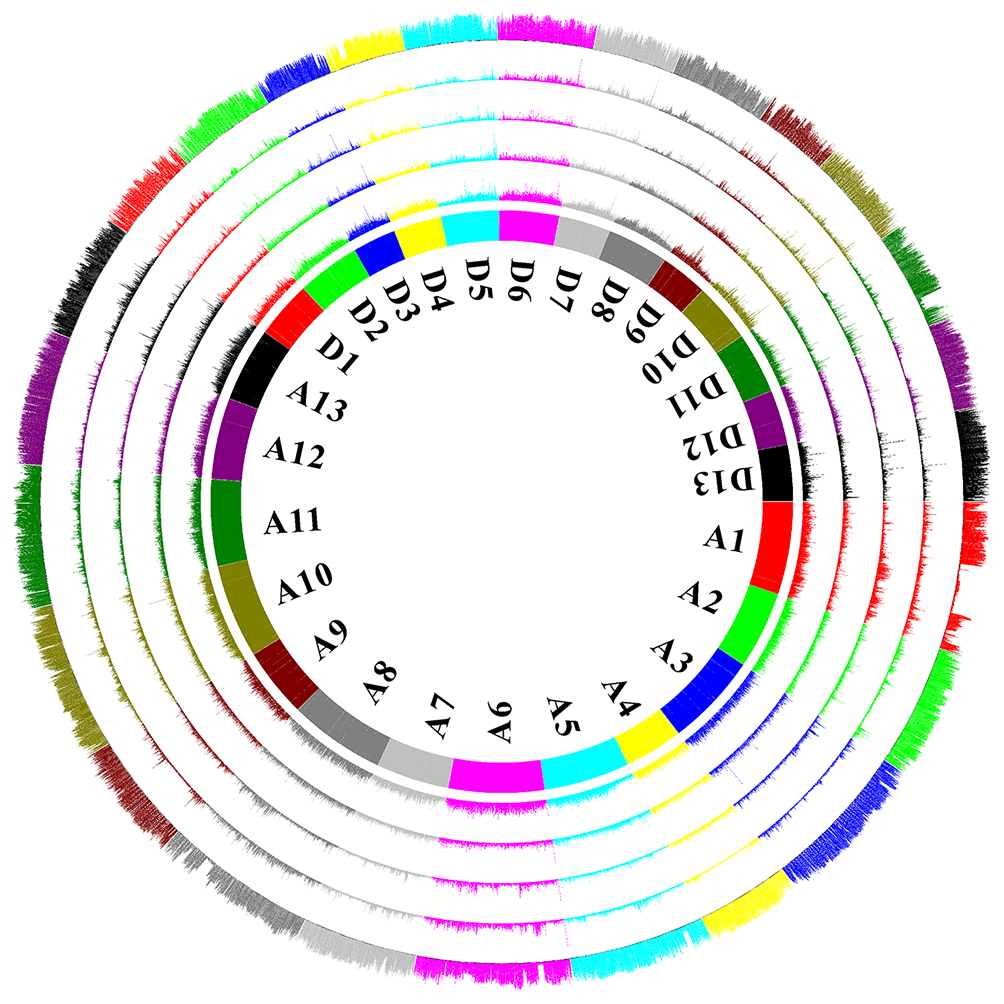


**Additional file 1: Figure S7. Characterization of 147 allotetraploid cotton genomes.** The 26 allotetraploid cotton chromosomes, A1 to D13, are represented by different colors. From the inner circle to the outer circle, the curves represent the SNP density, the InDel density, the level of genetic diversity in *G. hirsutum* (*π_Gh_*), the level of genetic diversity in *G. barbadense* (*π_Gb_*) and the level of genetic differentiation between *G. hirsutum* and *G. barbadense*, respectively.


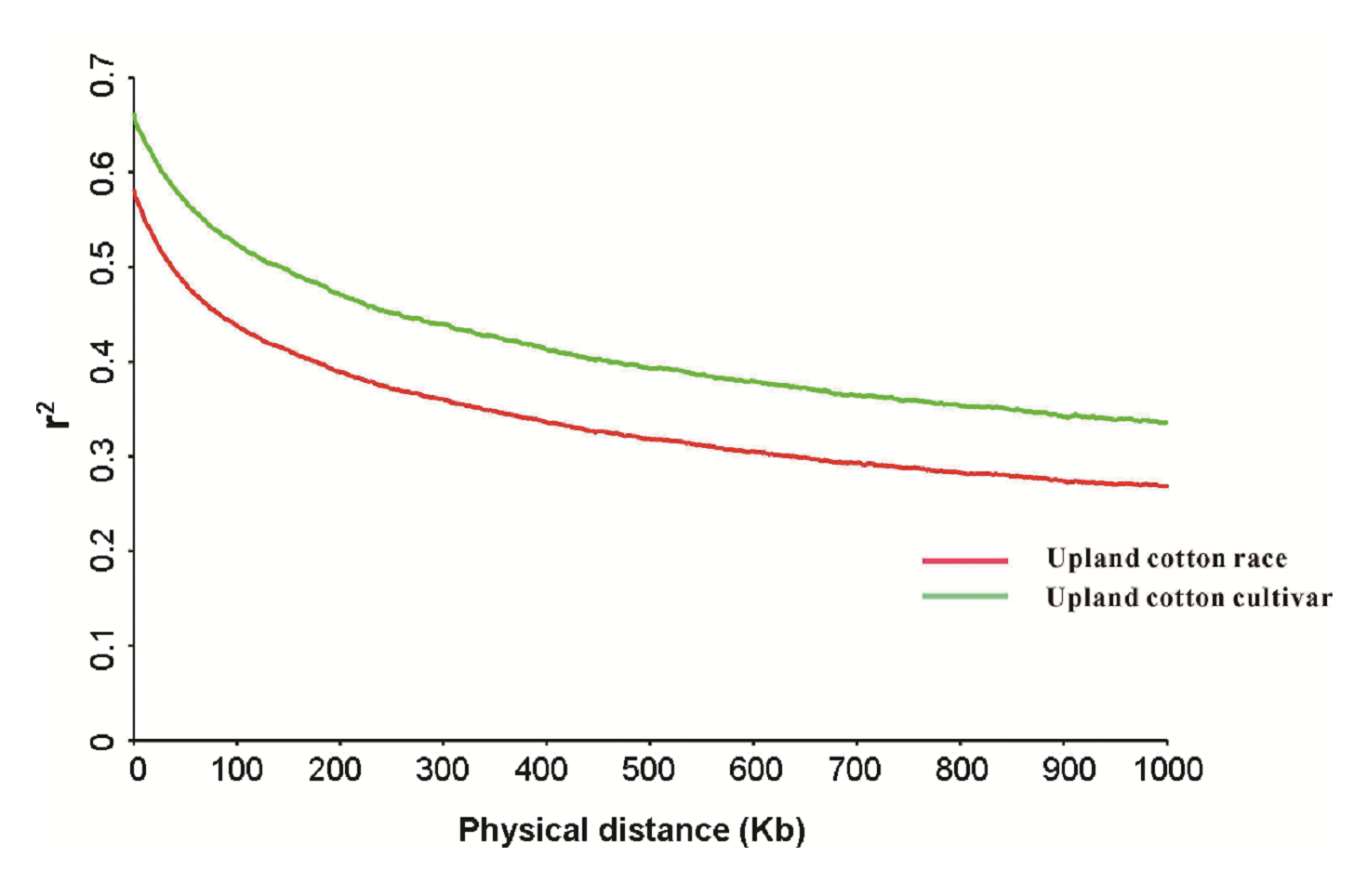


**Additional file 1: Figrue S8. Genome-wide average LD decay in *G. hirsutum* for races and cultivars, respectively.** LD was calculated using the software Haploview with default settings, and pairwise *r^2^* was calculated for all SNPs in a 1000-Kb window.


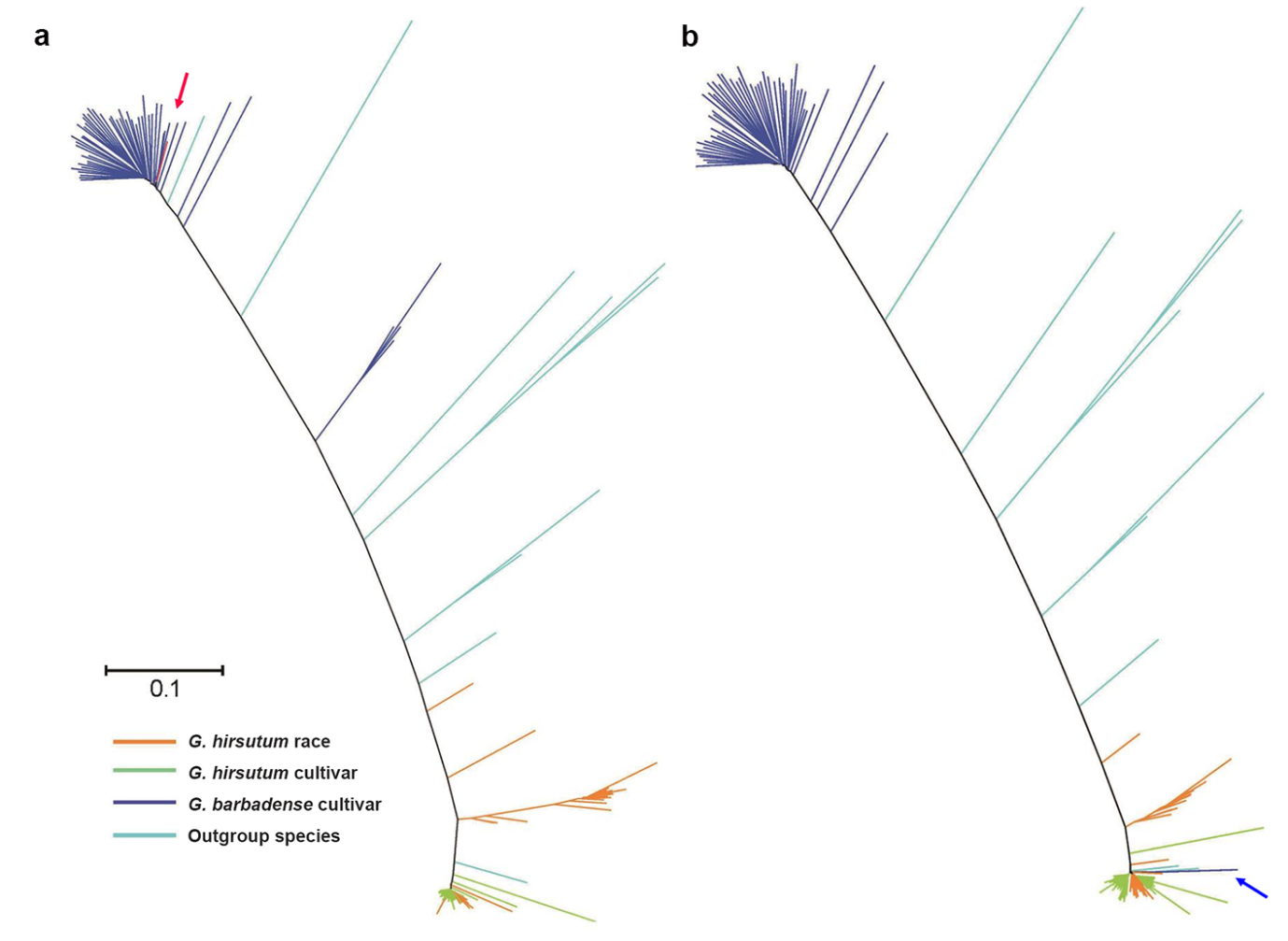


**Additional file 1: Figure S9. Phylogenetic tree of all cotton accessions in two introgression loci.** The cotton accession under introgression was indicated. (**a**) An introgression of *G. barbadense* alleles into *G. hirsutum* on chromosome A1 from 29 Mb to 30 Mb, shown as red arrow. (**b**) An introgression of *G. hirsutum* alleles into *G. barbadense* chromosome A2 from 47 Mb to 48 Mb, shown as blue arrow.


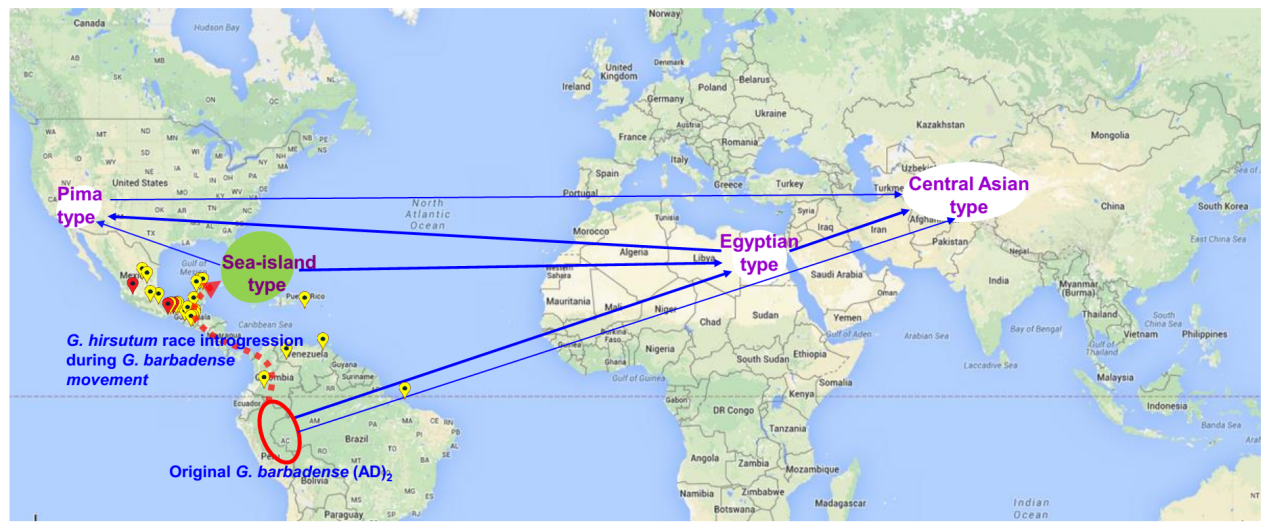


**Additional file 1: Figure S10. Domestication and improvement of *G. barbadense.*** Subsequent introgressions from the local *G. hirsutum* wild races into *G. barbadense* during its northern movement are shown. Such introgression might be responsible for the creation of the Sea Island cotton germplasm. The red marks indicated the three richimondi accessions and the yellow marks indicated the other races. The Egyptian-type ELS cottons were developed by crossing *G. barbadense* tree cotton, Jumel, with Sea Island to produce Ashmouni in Egypt. Ashmouni was introduced into the previous Soviet Union and used to further develop the Central Asia type ELS cottons. The Egyptian-type cottons were then reintroduced into America, integrated with the diverse germplasms from Sea Island cotton and American Upland cottons, and used to produce the Pima-type ELS cottons.


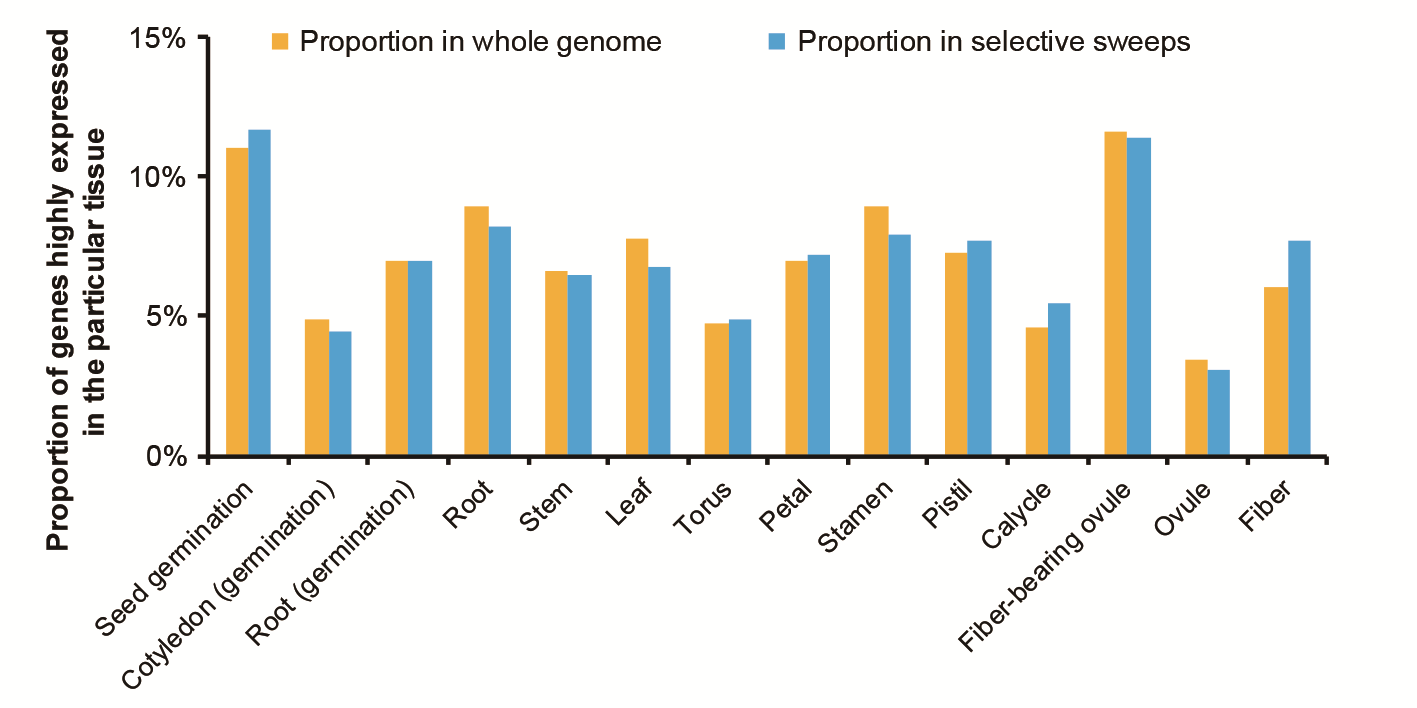


**Additional file 1: Figure S11. The gene expression profile is investigated for each gene, and transcription levels are quantified from the RNA-Seq data of 14 cotton tissues types (PRJNA248163).** The proportion of genes highly expressed in each tissue is displayed, for selective sweeps and whole genome, respectively. Seed germination includes cotyledon and hypocotyl after germination for 0, 5 and 10 hours.
