# Supplementary figures and images for: Genomic insights into divergence and dual domestication of cultivated allotetraploid cottons
Source: Genome Biol. 2017 Feb 20;18:33. doi: 10.1186/s13059-017-1167-5 (PMC5317056; doi:10.1186/s13059-017-1167-5)

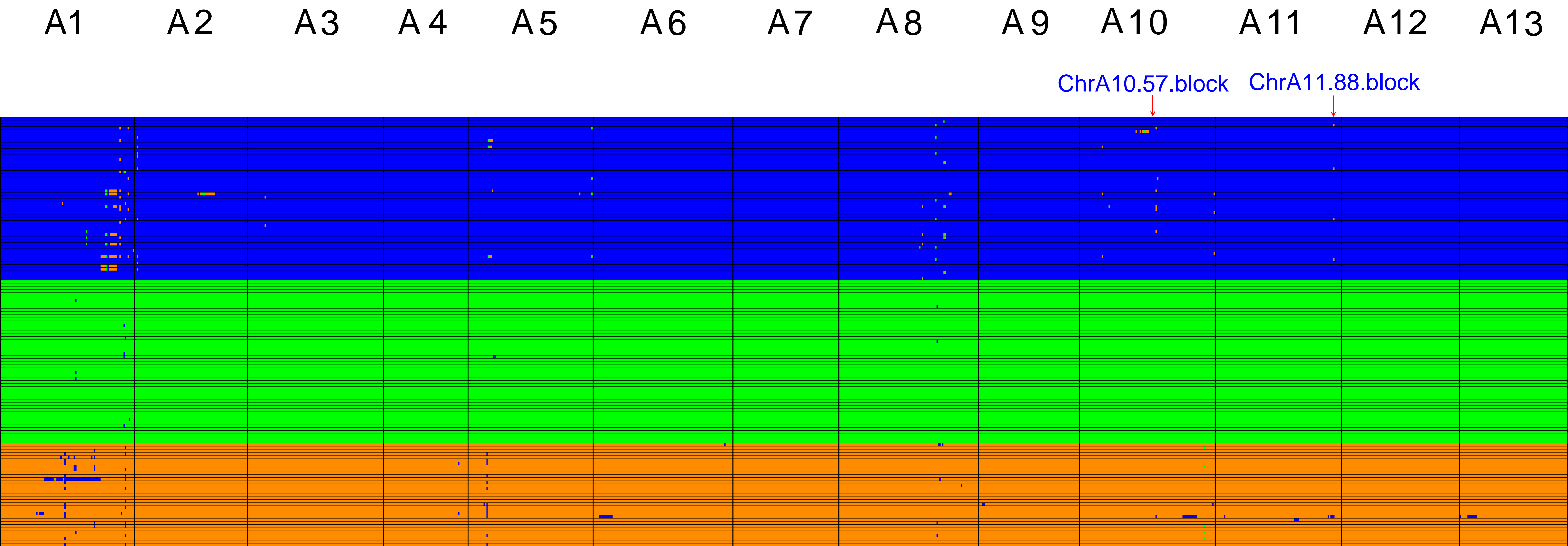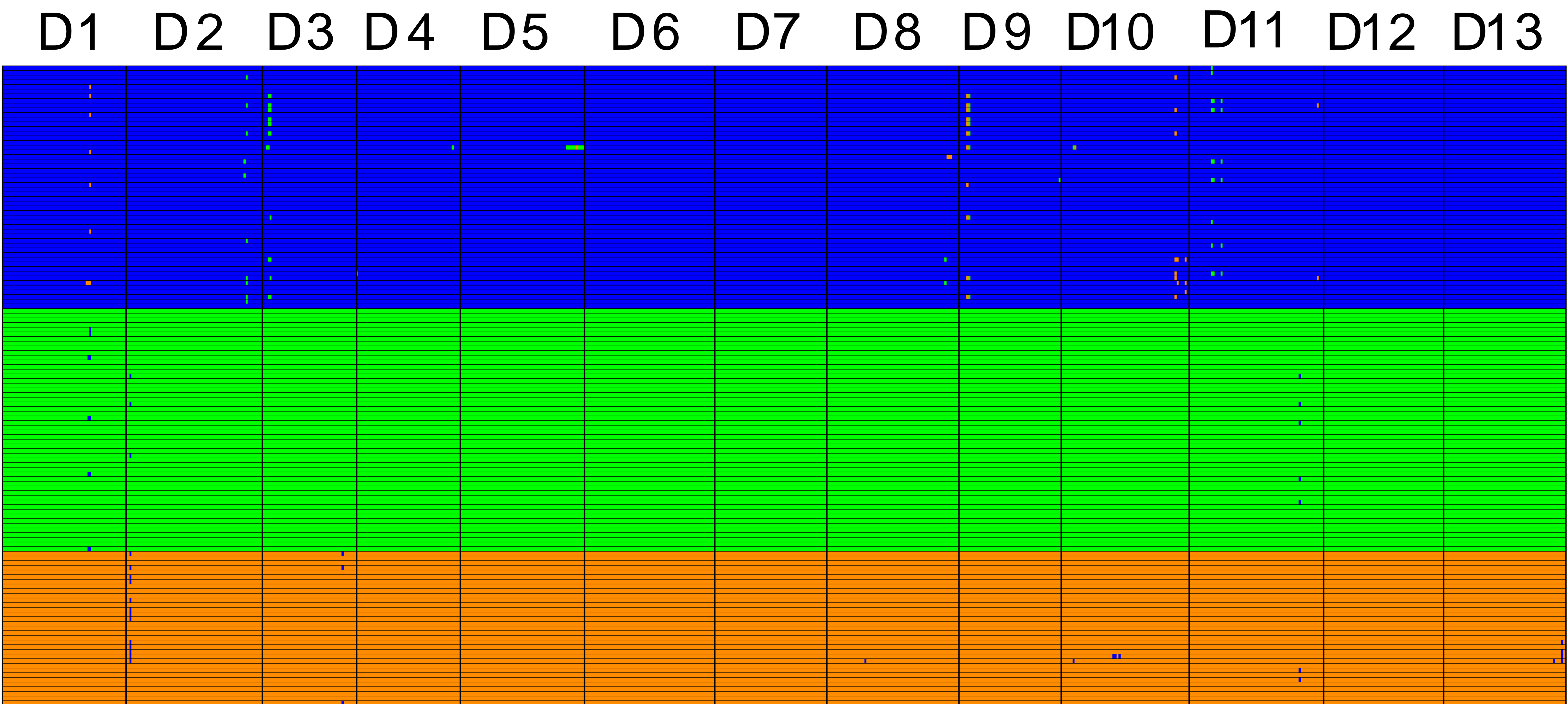

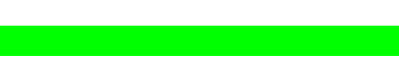 *G.hirsutum* cultivar  
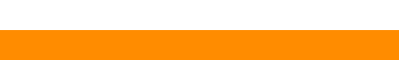 *G.hirsutum* race  
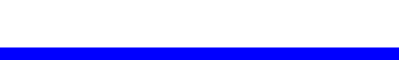 *G.barbadense* cultivar

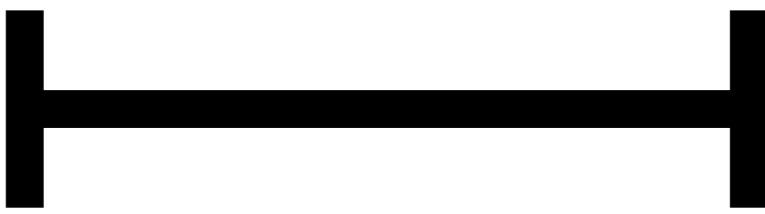 100MB

Supplement: Additional file 14: Dataset 2. — Whole-genome analysis of genetic introgressions in allotetraploid cotton. (PDF 111 kb) [file 13059_2017_1167_MOESM14_ESM.pdf]
