# Supplementary material for: Genomic insights into divergence and dual domestication of cultivated allotetraploid cottons
Source: Genome Biol. 2017 Feb 20;18:33. doi: 10.1186/s13059-017-1167-5 (PMC5317056; doi:10.1186/s13059-017-1167-5)

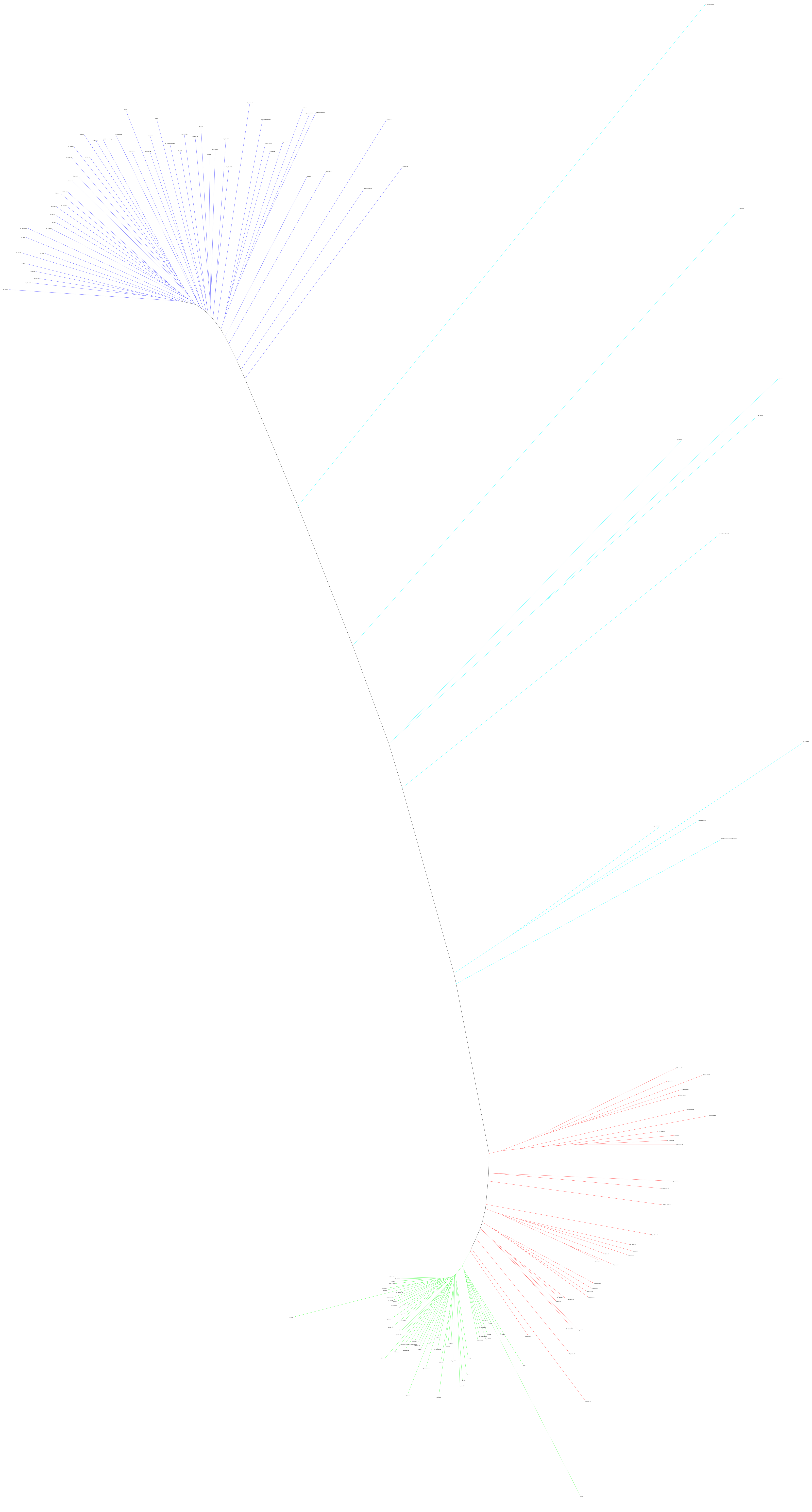

Supplement: Additional file 8: Dataset 1. — A phylogenetic tree with full accession names. (PDF 180 kb) [file 13059_2017_1167_MOESM8_ESM.pdf]
